# Genomic analysis of three *Bifidobacterium* species isolated from the calf gastrointestinal tract

**DOI:** 10.1038/srep30768

**Published:** 2016-07-29

**Authors:** William J. Kelly, Adrian L. Cookson, Eric Altermann, Suzanne C. Lambie, Rechelle Perry, Koon Hoong Teh, Don E. Otter, Nicole Shapiro, Tanja Woyke, Sinead C. Leahy

**Affiliations:** 1 AgResearch Limited, Grasslands Research Centre, Palmerston North, New Zealand; 2 Department of Energy, Joint Genome Institute, Walnut Creek, California 94598, USA

## Abstract

Ruminant animals contribute significantly to the global value of agriculture and rely on a complex microbial community for efficient digestion. However, little is known of how this microbial-host relationship develops and is maintained. To begin to address this, we have determined the ability of three *Bifidobacterium* species isolated from the faeces of newborn calves to grow on carbohydrates typical of a newborn ruminant diet. Genome sequences have been determined for these bacteria with analysis of the genomes providing insights into the host association and identification of several genes that may mediate interactions with the ruminant gastrointestinal tract. The present study provides a starting point from which we can define the role of potential beneficial microbes in the nutrition of young ruminants and begin to influence the interactions between the microbiota and the host. The differences observed in genomic content hint at niche partitioning among the bifidobacterial species analysed and the different strategies they employ to successfully adapt to this habitat.

Ruminant animals such as cattle and sheep contribute significantly to the global value of agriculture by supporting the livelihoods and food security of almost a billion people worldwide^1^. Ruminants are foregut fermenters that have evolved an efficient digestive system, which relies on a complex microbial community to ferment plant fibre and provide fermentation end-products and other nutrients for growth of the animal^2^. This reliance develops at birth where vertical transmission of microbes from the mother is considered a pivotal route for microbiota establishment in newborns^3^. Currently, little is known of the microbial-host interactions that occur during a ruminants’ early life and whether these interactions influence the lifetime performance and health of the animal.

Newborn ruminants (pre-ruminants) have a physically and metabolically underdeveloped rumen (fore-stomach) which means they are naturally dependent on milk during their early stages of life, and gradually transition and adapt to solid feed during which time the rumen develops^4^. The provision of milk to the neonate has considerable implications in the development of the infant intestinal microbiota. Milk selects for a highly adapted intestinal microbiota, dominated by bifidobacteria. Several constituents of milk such as oligosaccharides and glycoconjugates are known to selectively enrich for bifidobacteria^5^. These microbes have been shown to have a versatile and important role in human infant gut development ranging from stimulation and maintenance of the intestinal mucosal barrier and its immune response, to prevention of the attachment of pathogens and production of a range of beneficial metabolic substrates^6^,^7^,^8^. However, the on-farm management of pre-ruminants, particularly dairy calves, has traditionally focused on separating the newborn from their dams and restricting the amount of milk, or milk replacer, offered in order to encourage solid feed intake and accelerate weaning and rumen development^4^. This means calves do not have extended access to the nutritional and additional benefits that milk provides, and it remains to be seen whether this strategy has a negative impact on the animal’s development and subsequent performance. Recent research has shown that increased nutrient intake from milk, or milk replacer, during the pre-weaning period positively impacts lactation milk yield and lifetime performance of the animal^9^.

Pre-ruminants are often susceptible to a number of microbial pathogens during their first months of life which can severely affect growth efficiency and overall productivity^4^. Use of *Bifidobacterium* strains as direct-fed microbials (DFMs) are an attractive possibility that may provide beneficial effects for young livestock during a time when the risks of morbidity and mortality are high. There are several studies describing the isolation of bifidobacteria from young ruminants and some limited reports on their use as probiotics, which highlight their influence on immune function and their ability to reduce the incidence of diarrhea in young calves^10^,^11^. However, there is no research investigating the role of ruminant-derived bifidobacteria on ruminant gut development or whether there are beneficial microbial interactions occurring within the host ruminant during this early life phase. To begin to address these questions we have sequenced the genomes of three *Bifidobacterium* species isolated from the faeces of newborn calves and examined the ability of these organisms to grow on specific carbohydrates typical of the newborn ruminant diet.

## Results and Discussion

### Phylogenetic analysis

Three strains of *Bifidobacterium* were isolated from freshly expelled calf faecal samples during the milk-feeding period (1 to 5 days old). A phylogenetic analysis based on the 16S rRNA gene sequences of the type strains of the recognized species of the genus *Bifidobacterium* and the isolated strains from this study was performed (Fig. S1). This analysis indicated that the isolated strains were members of three different *Bifidobacterium* species; *Bifidobacterium choerinum* AGR2158, *Bifidobacterium pseudolongum* subsp. *globosum* AGR2145, and *Bifidobacterium longum* subsp. *suis* AGR2137. AGR2145 and AGR2158 were isolated from the same animal. *B. choerinum* was originally described as a species by Scardovi *et al.*^12^. It is considered an autochthonous *Bifidobacterium* species of the pig that is well adapted to the gut of pre-weaned piglets. However, *B. choerinum* has also been isolated from young ruminant faeces and from sewage^10^,^12^,^13^. Members of the *B. pseudolongum* species are typically isolated from the gastrointestinal tract with the subspecies *B. pseudolongum* subsp. *globosum* mainly found in the rumen and intestine of ruminants^14^. *B. longum* subsp. *suis* was defined as a subspecies by Mattarelli *et al.*^15^ alongside the two other subspecies *B. longum* subsp. *infantis* and *B. longum* subsp. *longum. B. longum* subsp. *suis* is commonly isolated from the faeces of piglets but has also been isolated from the faeces of young ruminants^13^. While members of the *Bifidobacterium* genus can be readily cultured from young ruminant sources, knowledge of their apparent abundance in the microbiome of the young ruminant has been limited. This has been a consequence of selected DNA extraction techniques, inadequate primer choice or a combination of both as evidenced by analysis of the human infant gut microbiota^16^,^17^. The strains isolated in this study are phylogenetically closely related to the *Bifidobacterium* lineages (*B. longum*, *B. animalis*, *B. breve,*) which are used and/or have high potential as probiotics in human infants^18^ (Fig. S1). This highlights their possible application as beneficial organisms for the development of the intestinal microbiota of pre-ruminants.

### General genome characteristics

**Figure 1:**
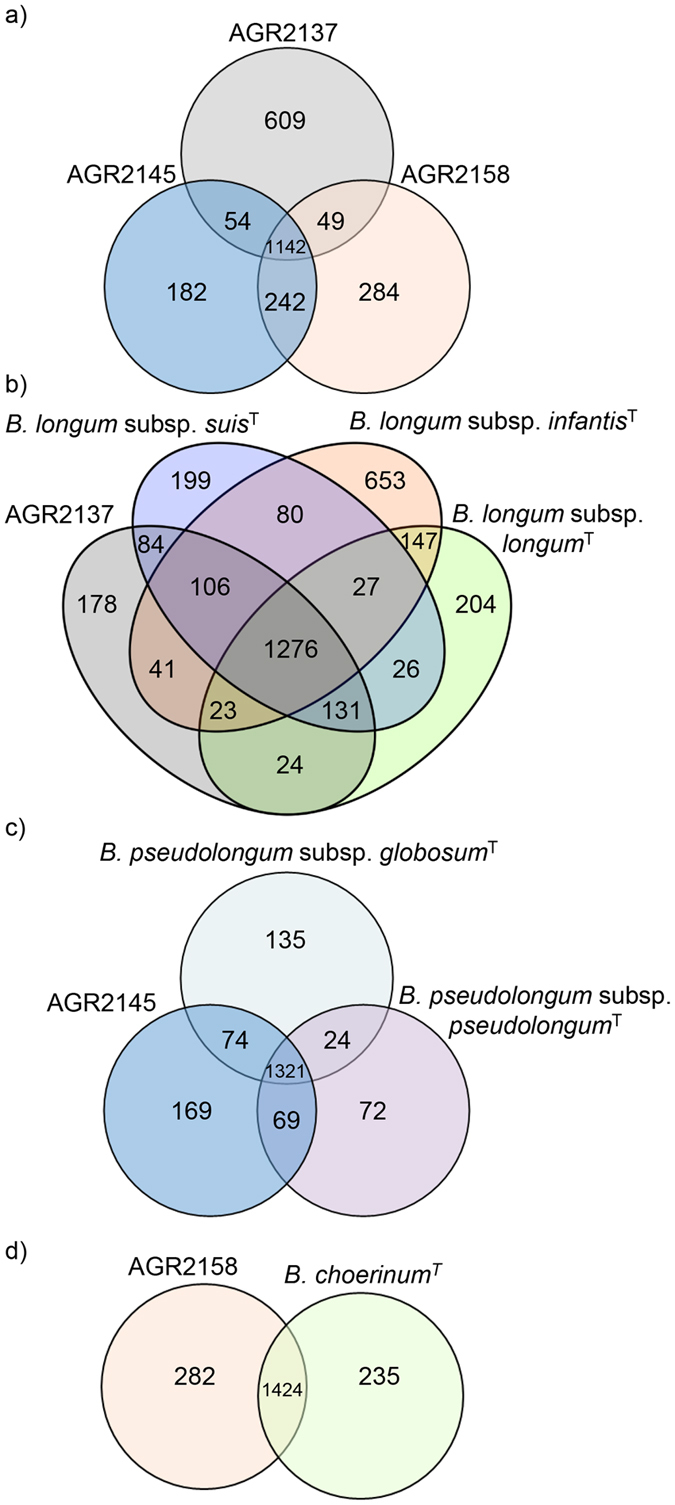
Genomic diversity of the calf bifidobacterial species. Panel a displays a Venn diagram of homologues shared between the three calf genome sequences. Panel b, c, & d show a Venn diagram representation of each of the respective calf genome sequences compared against the relevant type strain from the *Bifidobacterium* genus.

The genome features of the isolated strains and their comparison against relevant type strains of the genus *Bifidobacterium* are listed in Table 1. As expected, based on their phylogeny (Fig. S1) the genome sequences of the calf isolates AGR2145 and AGR2158 are more similar to each other than to AGR2137. Both genomes display a higher guanine and cytosine (G + C) content, 63.30% and 64.10% respectively, than that of the AGR2137 genome, 59.86%. These G + C percentages are consistent with what has been observed for the relevant type strains (Table 1)^19^. Additionally, both AGR2145 and AGR2158 display a more similar codon and amino acid usage profile than that of AGR2137 (Fig. S2). The three genomes range in size between 1.9 and 2.2 Mb which is within the range (1.73–3.25 Mb) reported for genome sequences of the genus *Bifidobacterium*^19^. Functional assignment of the calf bifidobacterial ORFeomes based on the Clusters of Orthologous Groups (COG) database indicate a functional designation for, on average, 67% of the total number of predicted protein-coding genes for each genome. A comparative analysis of the ORFeomes indicates a conserved set of 1,142 gene families shared between the three *Bifidobacterium* species examined. (Fig. 1a). COG categories were assigned to these 1,142 gene families. This analysis highlighted the comparable metabolism between the three calf isolates with 35% of the conserved gene families being assigned to metabolism, 23% to information storage and processing, 15% to cellular processes and processing and 20% to the poorly characterized category (Fig. S3). Genes unique to each of the calf genomes were examined and are discussed throughout the text (Figs 1 and S3). These genes may serve as targets for functional studies to elucidate the different strategies each of the calf bifidobacterial species employ to successfully adapt, co-exist, and survive within the pre-ruminant gastrointestinal tract. ORFeomes of the relevant type (sub) species were also compared against each of the relevant calf ORFeomes (Fig. 1b–d) to identify genes distinct to the calf isolates. As expected, the majority of these distinct genes (on average 67%) were assigned no functional annotation. Those assigned a functional category are summarised in Fig. S4 with the COG carbohydrate transport and metabolism category being the most abundant for the genomes of AGR2145 and AGR2137.

**Figure 2:**
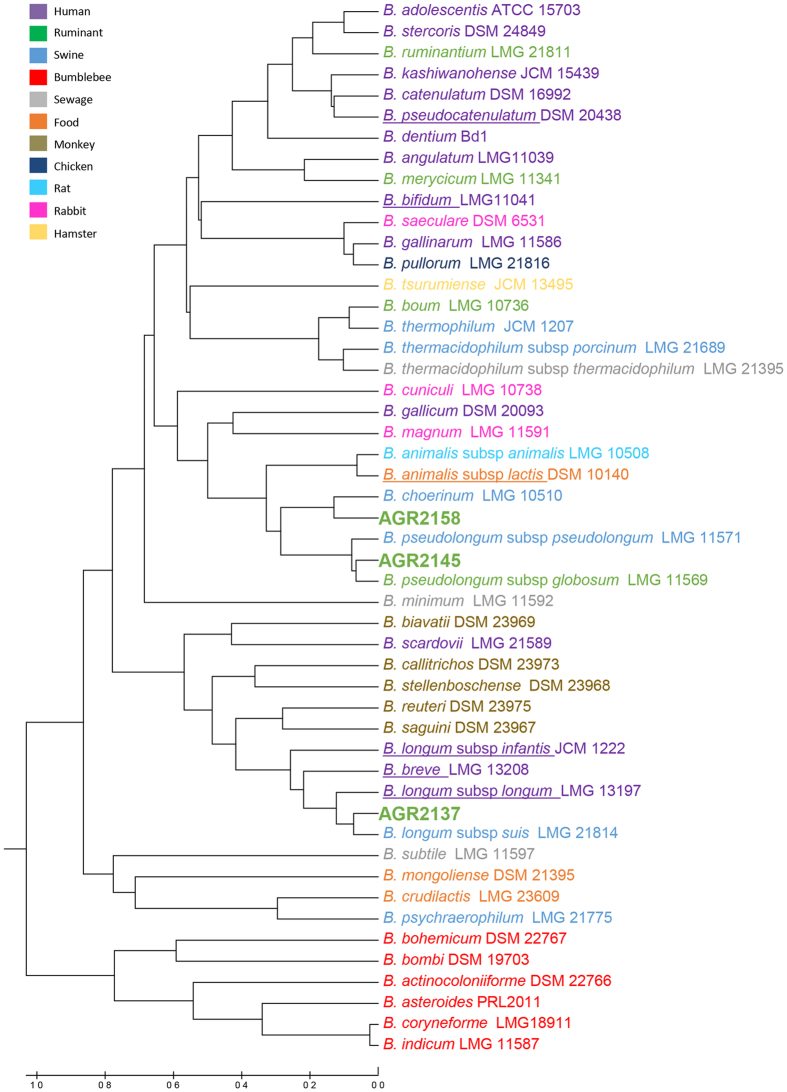
Functional genome distribution of the *Bifidobacterium* genus. All strains are colour-coded according to their isolation source and species with a use and/or high potential as probiotics in human infants, as detailed by Di Gioia *et al.*^18^, are underlined.

The availability of the genome sequences of members of the genus *Bifidobacterium* allowed a Functional Genome Distribution (FGD) tree to be constructed^20^ (Fig. 2). In contrast to an evolutionary phylogeny, FGD analyzes the functional relationship between microbes based on their predicted ORFeomes. This approach takes into account genotype adaptations which might render organisms more similar to each other than what their respective evolutionary heritage would indicate. Based on this analysis, the three genomes examined cluster closely with their respective type strains. The FGD tree is largely comparable with the phylogenetic clusters identified in the supertree described for the genus of *Bifidobacterium*^19^. Both AGR2145 and AGR2158 fall within the *B. pseudolongum* phylogenetic group and AGR2137 within the *B. longum* phylogenetic group. Analysis of potential horizontal gene transfer (HGT) events in the calf genome sequences identified percentages of alien genes, compared to the total number of ORFs. This analysis revealed 13.4% (AGR2137), 9.1% (AGR2145) and 7.8% (AGR21258) respectively, of the calf ORFeomes are predicted to have undergone HGT. Predicting the donors of these putative alien genes (Table S1) indicated a preferential origin from members of the *Alphaproteobacteria* for AGR2145 and AGR2158 while other members of the *Actinobacteria* were the preferential origin for AGR2137. Analysis of the COG assignment of these predicted alien genes, excluding genes with no known function, is summarised in Fig. S5.

Genomic features unique to the *B. choerinum* AGR2158 genome include a putative type VII/WXG100 secretion system and a prophage. A cluster of 15 open reading frames (ORFs) was identified in the genome of AGR2158 that represents a type VII/WXG100 secretion system (Fig. S6). This denotes the first report of such as system in the *Bifidobacterium* genus. However, comparative analysis has revealed a similar cluster of genes can also be found in the genome of *B. dentium* Bd1 (Fig. S6, Genbank accession number CP001750). Type VII/WXG100 secretion systems are known to be present in several Gram-positive organisms. They were initially identified in *Mycobacterium tuberculosis* and although type VII/WXG100 secretion systems are thought to play a role in bacterial pathogenesis in some organisms^21^ they have also been associated with other cellular functions^22^. Their role in bifidobacteria remains unknown. A 41 kb prophage (G606DRAFT_1765-1820) has been identified in the genome of AGR2158 (Fig. S7) which is different from the 56 kb prophage identified in the type strain of this species (LMG 10510)^23^. The modular organization of the AGR2158 prophage is similar to that reported for other *Bifidobacterium* prophages^24^,^25^.

### Genomics of carbohydrate utilization

Carbohydrate metabolism and transport is a major activity for bifidobacteria^26^,^27^ and genes involved in this functional category make up 9.5% (AGR2158), 11.8% (AGR2145) and 12.4% (AGR2137) of the calf *Bifidobacterium* genomes. The ability of the three strains to grow on various carbohydrates is shown in Table 2, and the genes predicted to encode proteins associated with carbohydrate metabolism and transport are shown in Table S2.

### Milk and host-derived carbohydrates

Studies of bifidobacteria from human infants have shown how strains of *B. bifidum* and *B. longum* subsp. *infantis* are able to metabolize human milk oligosaccharides^28^ and the glycan component of host mucins^29^. It is expected that bifidobacteria from young ruminants will be exposed to comparable nutrient sources, but this has not been investigated. Bovine milk and colostrum contain a diversity of free oligosaccharides, but their concentration is much lower than in human milk^23^. Bovine milk also contains several glycoproteins which are substituted with a range of N- and O-linked glycans^30^. The carbohydrate composition of calf intestinal mucin has been determined^31^ and is made up predominantly of galactose, N-acetylglucosamine, and N-acetylgalactosamine, with lower amounts of fucose, mannose and sialic acids. Ruminants also secrete large amounts of saliva which contains numerous N-linked glycoproteins^32^ but their carbohydrate composition is not known.

**Figure 3:**
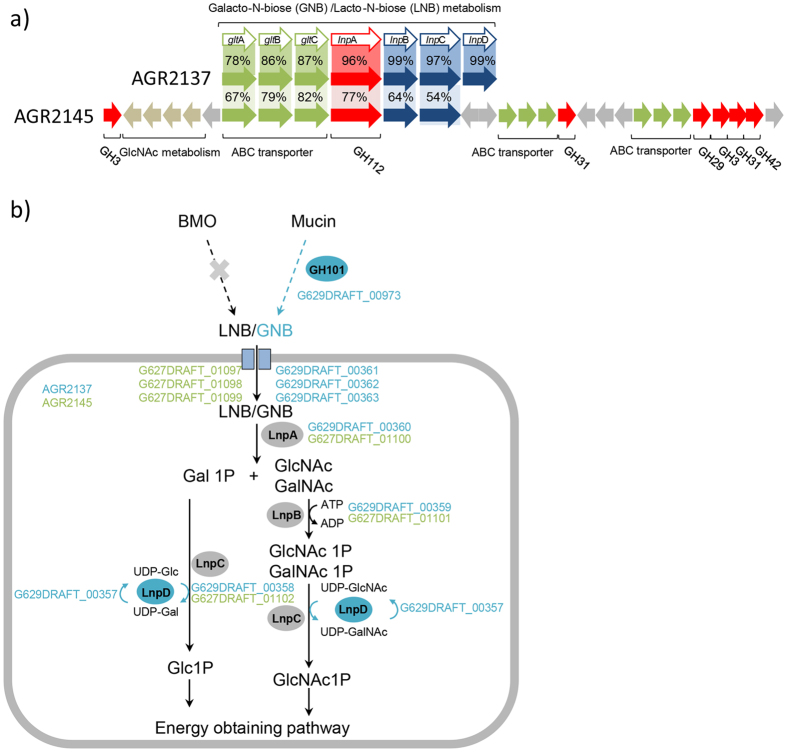
The galacto-N-biose (GNB)/lacto-N-biose (LNB) pathway found in bifidobacteria. (**a**) The gene cluster encoding the pathway was adapted from Kitaoka *et al.*^28^. Using the *B. longum* JCM1217 gene cluster (BLLJ_1620-1626)) as a reference (Genbank accession number NC_015067) the corresponding gene clusters are shown for AGR2137 (G629DRAFT_00357-00363) and AGR2145 (G627DRAFT_01097-01102). BLASTP percent identities are shown. Only genes predicted to be involved in the LNB/GNB gene cluster are shown for AGR2137 while additional carbohydrate utilising genes surrounding the LNB/GNB gene cluster are shown for AGR2145 (G627DRAFT_01091-01120). This diagram is not drawn to scale. (**b**) Schematic representation of the pathway. GalNAc1P, N-acetylgalactosamine 1 phosphate; Gal1P, galactose 1-phosphate; GlcNAc1P, N-acetylglucosamine 1 phosphate, Glc1P, glucose 1-phosphate. Locus tags of genes from AGR2137 are shown in blue, while those from AGR2145 are shown in green.

Lactose is the predominant carbohydrate in bovine milk and is able to be fermented by the three calf *Bifidobacterium* strains investigated (Table 2). Most bovine milk oligosaccharides are sialylated, but none of the calf strains were able to ferment 3′- or 6′-sialyl lactose, or had sialidase genes in their genome sequence. However, other bacteria in the gut may be able to initiate oligosaccharide breakdown and thus cross-feed the bifidobacterial strains^33^. Other bacteria isolated from the same samples as the three *Bifidobacterium* strains have also been genome sequenced and two of these bacteria (*Ruminococcus gnavus* AGR2154 and *Dorea longicatena* AGR2136) encode sialidases. The metabolism of lacto-N-biose (LNB) derived from milk oligosaccharides and galacto-N-biose (GNB) derived from mucin occurs via a common pathway and has been well studied in infant-derived bifidobacteria. AGR2137 encodes the gene cluster (Fig. 3a,b) for the complete LNB/GNB metabolism pathway as described in *B. longum*^34^. *B. pseudolongum* strains have previously been reported to ferment lacto-N-biose^35^ and AGR2145 encodes a similar gene cluster to that found in *B. longum* strains (G629DRAFT_00357-00363). However, in AGR2145 the *gal*E gene homolog (*lnp*D) is missing and the LNB/GNB genes form part of a novel 29 gene cluster ((G627DRAFT_01091-01120) which includes genes for two other ABC transporters and several glycoside hydrolases (Fig. 2a,b, Table S2). This gene cluster is not present in the genome of the type strain of *B. pseudolongum* subsp. *globosum* (DSM 20092^T^). The LNB/GNB metabolism genes are not part of the AGR2158 genome.

AGR2137 encodes homologues of glycoside hydrolases that have been shown to be involved in the metabolism of oligosaccharides derived from host mucins (Table S2). These include a GH101 family endo-alpha-N-acetylgalactosaminidase^36^ that cleaves GNB from gastroduodenal mucin, and an GH129 family alpha-N-acetylgalactosaminidase^37^ that removes N-acetylgalactosamine from intestinal mucin.

### Plant carbohydrates

The diet of forage fed ruminants is rich in structural carbohydrates including cellulose, hemicellulose and pectin, storage polysaccharides such as starch and water-soluble fructans and raffinose family oligosaccharides. Consequently, young ruminants are exposed to a variety of plant carbohydrates as they transition to having a fully functional rumen.

**Figure 4:**
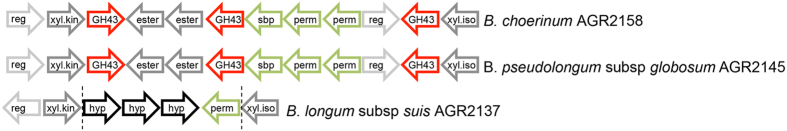
Genomic content and organization of XOS utilisation gene clusters. Gene functions are coloured as follows: glycoside hydrolases (GH) in red, carbohydrate esterases (ester), xylose isomerase (xyl.iso) and xylulose kinase (xul.kin) all in dark grey, ABC transporter substrate binding proteins (SBPs) and permeases (perm) in green, transcriptional regulators (reg) in light grey, hypothetical proteins (hypo) in black.

Hemicellulose is a major component of plant cell walls and its degradation results in the accumulation of xylo- and arabinoxylo-oligosaccharides (XOS, AXOS) that are selectively fermented by bifidobacteria. An AXOS utilization locus, bounded by xylose isomerase (*xyl*A) and xylulokinase (*xyl*B) genes, has been identified by transcriptional analysis in *B. animalis* subsp. *lactis*^38^ and a homologue of this locus is found in both AGR2145 and AGR2158 (Fig. 4). This locus includes the gene for a highly conserved ABC transporter substrate-binding protein which mediates the uptake of a range of XOS and AXOS oligomers^39^ (Table S2). In AGR2137 although the *xyl*A and *xyl*B genes are present, the other AXOS utilization locus genes have been lost (Fig. 4) and thus this strain is unable to ferment XOS (Table 2). Two of the calf *Bifidobacterium* strains are also able to grow on arabinose and xylose (Table 2). AGR2137 has genes for a pentose ABC transporter (G629DRAFT_00299-00304), while AGR2145 has a pentose ABC transporter as part of a larger gene cluster including a GH51 family alpha-L-arabinofuranosidase and genes for arabinose metabolism (G627DRAFT_00515-0000526). The pentose transporter has been lost from AGR2158 although a fragment of the permease gene remains (G606DRAFT_00824).

AGR2137 also encodes the gene cluster that mediates the utilization of galactans derived from pectin in several *B. breve* and B. *longum* strains (G629DRAFT_01106-01111)^40^. This locus contains the gene for a secreted GH53 family arabinogalactan endo-1,4-beta-galactosidase that acts on galacto-oligosaccharides (GOS) and releases galactotriose which is transported into the cell and further broken down by a GH42 family beta-galactosidase^40^.

*B. pseudolongum* strains have been shown to ferment starch, amylopectin and pullulan^41^, and both AGR2145 and AGR2158 encode several signal peptide-containing GH13 family glycoside hydrolases which may mediate the breakdown of dextran, pullulan, starch and other glucans. All three strains can ferment maltose (Table 2) and each encodes a maltose ABC transporter (COG2182) associated with GH13 and GH77 glycoside hydrolases (Table S2). The three calf strains ferment fructo-oligosaccharides (FOS, Table 2) and encode a three gene operon similar to that shown to be involved in fructo-oligosaccharide breakdown in *B. breve* and *B. longum*^42^. A sucrose utilization operon containing a GH13 sucrose phosphorylase/transglycosylase has been characterized in *B. longum*^43^ and is present in AGR2137 and AGR2158. However, AGR2145 is unable to use sucrose (Table 2) and the operon in this strain (G627DRAFT_00811-00815) contains an inserted toxin-antitoxin gene cassette (Table S2).

Melibiose and raffinose are found in a wide variety of plants and a cluster of genes containing the alpha-galactosidase necessary to metabolize these sugars has been characterized in bifidobacteria^44^. AGR2145 and AGR2158 have a gene cluster similar to that found in *B. animalis* strains^37^,^44^, with genes encoding two GH36 and one GH13 glycoside hydrolases, while AGR2137 has a different gene arrangement with only one GH36.

### Microbial-host interactions

During its lifetime, a ruminant will continuously encounter microorganisms that range from those essential for health and productivity to those causing disease, hampering productivity. Consequently, the ruminant’s immune system must learn to distinguish between beneficial and pathogenic microbes, and understanding how this is mediated will be important to improve the lifetime performance of the animal.

**Figure 5:**
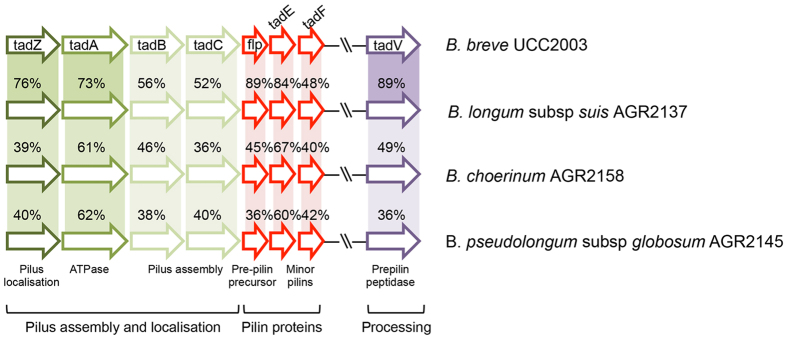
Schematic representation of the tad locus. Gene names and the functions of the encoded proteins are indicated. Figure adapted from O’Connell Motherway *et al.*^8^.

Microbial surface appendages are considered important for microbial-host interactions, and several species of bifidobacteria are known to produce pili-like structures^45^. Research with *B. breve* UCC2003 identified a type IVb tight adherence (Tad) pilus-encoding gene cluster which is essential for efficient *in vivo* murine gut colonization, and supports the concept of a pili-mediated host colonization and persistence mechanism for bifidobacteria^8^. The genomes of the three calf strains all contain a homologous tad locus (Fig. 5) suggesting that type IVb pili may play a role as a host colonization factor in the gastrointestinal tract of young ruminants. Members of the *Bifidobacteriaceae* are also known to elaborate pili via a sortase-assembly mechanism. Sortase dependent pili of *B. bifidum* PRL2010 have been shown to modulate bacterium-host interactions^46^,^47^. Analysis of the genomes of AGR2158 and AGR2145 has identified two sortases in each genome that cluster with genes encoding proteins with predicted cell wall sorting signals, and which may represent pilus loci (Fig. S7). Further investigation will be necessary to determine any involvement in host interactions.

Exopolysaccharides (EPS) are considered surface molecules that act as mediators of cross-talk between microbes and their host. Several beneficial activities have been attributed to EPS synthesized by *Bifidobacterium* species including modulation of the host’s immune system, formation of a protective physical barrier, modulation of the intestinal microbiota, and antagonism against pathogens^48^. The genomes of AGR2145 and AGR2158 each encode two EPS gene clusters (Table S3) which correspond to the relatively conserved *eps*3 and *eps*4 gene clusters which have been described as being common to strains of *B. pseudolongum*, *B. choerinum* and *B. animalis* isolated from the gastrointestinal tract of animals^49^. The EPS genes were not assembled in the AGR2137 genome (Table S3), but rhamnose biosynthesis genes which are a common feature of bifidobacterial EPS gene clusters are present^49^. Future work will be required to confirm EPS production in the strains analysed.

Bile salts play an important role in the host’s defence against ingested microorganisms^50^, and constitute a physiological barrier, which bifidobacteria need to overcome in order to survive and colonize within the gastrointestinal tract of the young ruminant. Specific bile resistance mechanisms reported in bifidobacteria include bile salt hydrolysis^51^ and bile efflux^52^,^53^. Bile salt hydrolases (BSHs) also referred to as cholylglycine hydrolases catalyse the hydrolysis of glycine- and/or taurine-conjugated bile salts into amino acid residues and free bile acids. The genomes of all three calf isolates contain a *bsh* gene (AGR2137, G629DRAFT_01360; AGR2145, G627_00122; AGR2158, G606DRAFT_1516). Genetic organisation around the *bsh* gene is analogous to that reported for *B. animalis* (AGR2145 and AGR2158) or *B. longum*^51^ (AGR2137). Efflux pumps are a common detoxification mechanism employed by bacteria and their role in bile tolerance has been demonstrated for *B. breve* and *B. longum*^52^,^53^. The AGR2137 genome has a homologous bile efflux pump gene (G629DRAFT_00578) and transporter gene (G629DRAFT_00581) suggesting a common mechanism of bile resistance in these species.

Fukuda *et al.*^7^ identified a ‘probiotic’ carbohydrate ABC transporter in three *Bifidobacterium* strains that correlated with the ability of these strains to protect against *E. coli* O157-induced death in mice. Expression of these transporter genes was highly induced by fructose, and it was proposed that fructose metabolism enables these cells to produce sufficient acetate to inhibit *E. coli*. This ABC transporter is characterized by the presence of genes assigned to functional categories COG1129, COG1172 and COG1879. The same gene cluster is found in AGR2137 (G629DRAFT_00719-00722), and this was the only calf strain able to use fructose (Table 2).

## Conclusion

This study has examined the genomes of three species of *Bifidobacterium* isolated from newborn calves, and focused on the genetic strategies these bacteria use for carbohydrate metabolism and host colonization and persistence. Dietary carbohydrates and host-derived glycans are the main energy sources for bifidobacteria and all three strains have the ability to utilize a range of carbohydrates that are likely to be available in the calf gastrointestinal environment. However, *B. longum* subsp. *suis* AGR2137 seems better adapted to the utilization of host glycans, while *B. pseudolongum* subp. *globosum* AGR2145 may have a more developed repertoire of genes allowing for the utilization of milk oligosaccharides, and *B. choerinum* AGR2158 looks to be restricted to the utilisation of plant polysaccharides. These differences in carbohydrate preference, and the presence of additional carbohydrate metabolism genes (such as sialidases) in other bacteria from the same environment, highlight the cooperative nature of polysaccharide metabolism and cross-feeding in gut environments. Analysis of the genomes has also provided insights into the association of these bifidobacterial species with their hosts and has resulted in the identification of several genes with a potential role in these processes. AGR2137 appears to be more similar in its predicted host-microbial processes to those described for the human infant related bifidobacterial species. The differences in genomic content hint at niche partitioning among the three different bifidobacteria analysed and give a first indication of the different strategies they employ to successfully colonize and survive in the pre-ruminant gastrointestinal habitat.

## Methods

### Isolation and 16S rRNA gene sequencing

Freshly voided faecal samples were taken from calves (1 to 3 days old) using a sterile wooden spatula and placed in a pre-weighed plastic container. Samples were weighed again, resuspended in 1 ml MS buffer and serially diluted in MS buffer under anaerobic conditions. MS buffer contained 40 ml mineral solution 1 (6 g/L K_2_HPO_4_), 40 ml mineral solution 2 (6 g/L KH_2_PO_4_, 6 g/L (NH4)_2_SO_4_, 12 g/L NaCl, 2.55 g/L MgSO_4_.7H_2_O, 1.69 g/L CaCl_2_.2H_2_O), 500 μl resazurin (0.1% w/v) and distilled water to 1 L. The solution was boiled and cooled under O_2_-free N_2_ and pH adjusted to 6.9, and then 0.5 g cysteine.HCl and 3 g Na_2_CO_3_ were added. The serially diluted faecal suspensions (90 μl volumes) were plated in duplicate onto bifidobacterial selective medium made according to the method of Leuscher *et al.*^54^ and incubated at 37 °C in an anaerobic glove box (95% CO_2_, 5% H_2_). Plates were inspected after 24 and 48 hrs and individual colonies were purified by repeated streaking onto fresh agar plates. Colony purity was confirmed by Gram stain and microscopic examination. Bacterial strains were stored at −85 °C on de Man, Rogosa, Sharpe (MRS, Oxoid) or Reinorced Clostridial Medium (RCM, Oxoid) agar slopes.

Full length amplification and sequencing of the 16S rRNA gene from the calf bifidobacterial isolates was undertaken using the bifF11 (5′-AGG GTT CGA TTC TGG CTC AGG), bifF782 (5′-GAT TAG ATA CCC TGG TAG TCC), bifR711 (5′-TTC CCG ATA TCT ACA CAT TCC), and bifR1543 (5′-AGG TGA TCC AGC CGC ACC TTC C) primers, designed to match conserved regions of the bifidobacterial full length 16S rRNA gene determined using an alignment of twenty diverse species generated in MEGA6^55^. PCR amplicons were generated using the KAPA Readymix HiFi HotStart ReadyMix according to the manufacturer’s instructions. After column purification (Qiagen PCR purification kit) PCR products were Sanger sequenced (Massey Genome Service, Massey University, New Zealand) and full length contiguous sequences assembled in Geneious (version 7.0).

### Carbohydrate growth assays

Bifidobacterial cultures were routinely cultured in MRS broth supplemented with 0.05% (w/v/) cysteine HCl. MRS media was dissolved in distilled water, boiled and then cooled on ice under a stream of O_2_-free CO_2_. Once cooled the cysteine HCl was added and aliquots were dispensed into Hungate tubes gassed with CO_2_. For carbohydrate utilisation a semi-synthetic medium based on MRS was used that was supplemented with carbohydrate (1% w/v) immediately before inoculation. The carbohydrates added were glucose, fructose, galactose, xylose, arabinose, N-acetyl galactosamine (Sigma), sucrose, cellobiose, lactose, melibiose (Sigma), maltose, 3′ or 6′ sialyl lactose (Carbosynth, UK), raffinose (Sigma), inulin (average DP ≥23, Orafti HP, Beneo Orafti, Belgium), galacto-oligosaccharide (Neo Cremar Co., Ltd., South Korea), fructo-oligosaccharide (DP 2–8, Orafti P95, Beneo Orafti, Belgium), xylo-oligosaccharide (DP 2–8, Shandong Longlive Biotechnology Co., Ltd., China). Each carbohydrate was made as a 10% (w/v/) stock solution in distilled water, and sterilised by filtration through a 0.22 μm syringe filter into a rubber-stoppered serum bottle that had been flushed with O_2_-free N_2_ and autoclaved. Stationary phase bifidobacterial cultures (100 μl) were inoculated aseptically into carbohydrate-supplemented broth using a sterile syringe filled with O_2_-free CO_2_ and the optical density at 600nm recorded immediately. Carbohydrate utilisation was assessed in triplicate with A_600_ measurements recorded over a period of 24–36 hours. Without carbohydrate supplementation, the semi-synthetic medium was unable to sustain bacterial growth above an A_600_ of ~0.2.

### Genomic DNA preparation

Cultures of *Bifidobacterium* strains AGR2137, AGR2145 and AGR2158 were grown in Reinforced Clostridial Medium (RCM) at 37 °C under anaerobic conditions. DNA for sequencing was obtained using the Qiagen Genomic-tip kit and following the manufacturer’s instructions for the 500/g size extraction.

### Genome sequencing and sequence analysis

The draft genomes of strains AGR2145, AGR2158 and AGR2137 were generated at the Joint Genome Institute using Illumina technology. For each of the genomes, an Illumina standard shotgun library was constructed and sequenced using the Illumina HiSeq 2000 platform. Annotation and analysis of the genomes was performed using a combination of the Integrated Microbial Genomes (IMG) Expert Review system^56^ and a manual curation using a GAMOLA/ARTEMIS software suite as described previously^57^. To identify conserved gene families, the program OrthoMCL (v1.4) was used using default parameters^58^. To identify genes that may have been acquired by horizontal gene transfer (HGT), the results from Alienhunter^59^ and COLOMBO (v3.8) implemented with the program SIGI-HMM^60^ were merged. Alienhunter was run with default parameters, while COLOMBO was run with a sensitivity value of 0.4. To generate the FGD tree, draft and completed *Bifidobacterium* (type strains) genomes^19^ in FASTA format were downloaded from the National Center for Biotechnology (NCBI). Draft genomes were concatenated using a non-bleeding spacer sequence and gene models were created for all genomes using an updated version of GAMOLA^57^. Resulting Genbank files were then subjected to a Functional Genome Distribution (FGD) analysis^20^ and the calculated distance matrix was imported into Molecular Evolutionary Genetics Analysis version 6 (MEGA6)^55^. The functional distribution was visualized using the unweighted pair group method with arithmetic mean (UPGMA).

## Additional Information

**How to cite this article**: Kelly, W. J. *et al.* Genomic analysis of three *Bifidobacterium* species isolated from the calf gastrointestinal tract. *Sci. Rep.* **6**, 30768; doi: 10.1038/srep30768 (2016).

## Supplementary Material

Supplementary Information

## Figures and Tables

**Table 1:** General features of the *Bifidobacterium* strains used in this study.

Organism	*B. longum* subsp. *suis*	*B. longum* subsp. *suis*	*B. longum* subsp. *infantis*	B*. longum* subsp. *longum*	*B. pseudolongum* subsp. *globosum*	B*. pseudolongum* subsp. *globosum*	B*. pseudolongum* subsp. *pseudolongum*	B. *choerinum*	*B. choerinum*
Strain	AGR2137	LMG21814^T^	ATCC15697^T^	LMG13197^T^	AGR2145	LMG11569^T^	LMG11571^T^	AGR2158	LMG10510^T^
Isolation source	Calf faeces	Pig faeces	Infant intestine	Adult intestine	Calf faeces	Bovine rumen	Pig faeces	Calf faeces	Piglet faeces
Status	Draft	Draft	Complete	Draft	Draft	Draft	Draft	Draft	Draft
Number of DNA scaffolds	49	36	1	8	30	26	11	19	20
Genome size (bp)	2,270,377	2,335,832	2,832,748	2,384,703	1,992,204	1,935,255	1,898,683	2,190,080	2.096,121
G + C content (%)	59.86	59.96	59.86	60.33	63.30	63.39	63.06	64.10	65.53
Number of protein-coding genes	1,897	1,999	2,486	1,924	1,654	1,598	1,551	1742	1,684
Number of genes assigned to COGs	1,250	1,253	1,396	1,235	1,119	1,089	1,072	1,143	1107
Number of genes with signal peptides	85	57	133	67	88	48	48	100	52
Number of genes with transmembrane helices	497	517	627	509	441	417	416	467	448
Genbank accession no.	ATWX00000000	JGZA00000000	CP001095	JGYZ00000000	ATWW00000000	JGZG00000000	JGZH00000000	AUJM00000000.1	JGYU00000000

**Table 2:** Carbohydrate utilisation by the three *Bifidobacterium* strains.

Carbohydrate	AGR2137	AGR2145	AGR2158
Arabinose	+	+	−
Fructose	+	−	−
Galactose	+	+	+
Glucose	+	+	+
N-acetyl glucosamine	−	−	−
Xylose	+	+	−
Cellobiose	−	−	−
Lactose	+	+	+
Maltose	+	+	+
Melibiose	+	+	+
Sucrose	+	−	+
3′-sialyl lactose	−	−	−
6′-sialyl lactose	−	−	−
Raffinose	+	+	+
Fructo-oligosaccharides	+	+	+
Galacto-oligosaccharides	+	+	+
Inulin	−	−	−
Xylo-oligosaccharides	−	+	+
