# Supplementary material for: Genomic analysis of three Bifidobacterium species isolated from the calf gastrointestinal tract
Source: Sci Rep. 2016 Jul 29;6:30768. doi: 10.1038/srep30768 (PMC4965825; doi:10.1038/srep30768)
Supplement: Supplementary Information [file srep30768-s1.pdf]

Genomic analysis of three Bifidobacterium species isolated from the calf gastrointestinal tract.

William J Kelly[1#], Adrian L Cookson[1#], Eric Altermann[1], Suzanne C Lambie[1], Rechelle Perry[1], Koon Hoong Teh[1], Don E Otter[1], Nicole Shapiro[2], Tanja Woyke[2], Sinead C Leahy[1*].

[1]AgResearch Limited, Grasslands Research Centre, Palmerston North, New Zealand.

[2]Department of Energy, Joint Genome Institute, Walnut Creek, California 94598, USA.

[#]Both authors contributed equally

*To whom correspondence should be addressed. Tel. +64 6 3518048. Fax +64 3518032. Email: Sinead.leahy@agresearch.co.nz

**Supplementary Legends**

**Table S1.** Putative donors of the predicted HGT in the calf bifidobacterial genomes

**Table S2.** Open reading frames (ORFs) predicted to encode proteins involved in carbohydrate metabolism and transport.

**Table S3.** Scaffold information and locus tag numbers of exopolysaccharide (EPS) gene clusters in the AGR2137, AGR2145 and AGR2158 genomes.

**Figure S1.** Phylogenetic tree derived from 16S rRNA gene sequences showing the position of the three strains sequenced in this study relative to the type strains of the genus *Bifidobacterium*. All strains are colour-coded according to their isolation source and strains with a use and/or have high potential as probiotics in human infants (Di Gioia et al., 2014) are underlined. The evolutionary history was inferred by using the Maximum Likelihood method based on the Tamura-Nei model. The tree with the highest log likelihood (-6411.3667) is shown. Initial tree(s) for the heuristic search were obtained automatically by applying Neighbor-Join and BioNJ algorithms to a matrix of pairwise distances estimated using the Maximum Composite Likelihood (MCL) approach, and then selecting the topology with superior log likelihood value. A discrete Gamma distribution was used to model evolutionary rate differences among sites (5 categories (+G, parameter = 0.1269)). The rate variation model allowed for some sites to be evolutionarily invariable ([+I], 70.3951% sites). The tree is drawn to scale, with branch lengths measured in the number of substitutions per site. The analysis involved 52 nucleotide sequences. All positions with less than 95% site coverage were eliminated. That is, fewer than 5% alignment gaps, missing data, and ambiguous bases were allowed at any position. There were a total of 1124 positions in the final dataset. Evolutionary analyses were conducted in MEGA6 (Tamura et al., 2013). This tree is consistent with the 16S rRNA gene tree that has been described for *Bifidobacterium* (Lugli et al., 2014)

**Figure S2.** Codon (a) and amino acid usage (b) of the draft genomes of AGR2137, AGR2145 and AGR2158. This analysis was performed using CMG-Biotools (Vesth et al., 2013)

**Figure S3.** COG functional classification of the 1, 142 conserved gene families (blue) identified in an OrthoMCL comparison of the ORFeomes of AGR2158, AGR2145 and AGR2137. From this comparison, genes unique to each genome have also been assigned a COG classification and are shown. This analysis relates to Fig 1a in the main manuscript.

**Figure S4** COG functional classification of the genes identified as distinct to each calf bifidobacterial genome as compared to the type strain of the respective species. Genes assigned to the COG poorly characterized categories (R, S and No hit) are excluded. This analysis relates to Fig 1a, b & c in the main manuscript.

**Figure S5.** COG functional classification of the predicted alien genes from each calf bifidobacterial genome sequence. Genes assigned to the COG poorly characterized categories (R, S and No hit) are excluded.

**Figure S6.** Genes encoding the putative type VII/WXG100 secretion system in AGR2158 (a), proposed cell membrane organization of the proteins predicted to be encoded by these genes (b), and the homologous genes in the *B. dentium* Bd1 genome (c).

**Figure S7.** Genomic organisation of the putative prophage in AGR2158. ORFs are drawn to scale and annotations are shown in vertical text.

**Figure S8.** Sortases in the genomes of AGR2145 and AGR2158 that cluster with genes encoding proteins with predicted cell wall sorting signals, and which may represent pilus loci.

**Table S1.** Putative donors of the predicted HGT in the calf bifidobacterial genomes

| **Table S1**. Putative donors of the predicted HGT in the calf bifidobacterial genomes | | | |
|---|---|---|---|
| **Putative Donor** | ***AGR2158 %** | ***AGR2145 %** | ***AGR2137 %** |
| *Alphaproteobacteria* | 72.7 | 45.8 | 14.3 |
| *Halobacteria* | 9.1 | 2.8 | |
| *Gammaproteobacteria* | 9.1 | 14 | 29.2 |
| *Flavobacteria* | 3.9 | 6.9 | 9.5 |
| *Actinobacteria* | 1.3 | 9.7 | 30.4 |
| *Betaproteobacteria* | 1.3 | 13.8 | 4.2 |
| *Chlorobia* | 1.3 | 2.8 | 2.4 |
| *Deltaproteobacteria* | 1.3 | 1.4 | 5.4 |
| *Sphingobacteria* | | 1.4 | |
| *Bacilli* | | 1.4 | 2.4 |
| *Chloroflexi* | | | 0.6 |
| *Bacteroides* | | | 0.6 |
| *Nitrospira* | | | 0.6 |
| *Spirochaetes* | | | 0.6 |
| *The percentage of the total HGT identified for each genome corresponding to the various taxa | | | |

**Table S2.** Open reading frames (ORFs) predicted to encode proteins involved in carbohydrate metabolism and transport.

A. *Bifidobacterium longum* subsp. *suis* AGR2137.

| Locus tag G629DRAFT | Gene name | Comments† | Accession No. | Protein identity | References |
|---|---|---|---|---|---|
| _00001 | sugar ABC transporter permease | | | | |
| _00002 | glycoside hydrolase GH42 family | beta-galactosidase/transgalactosylase | AAU00556 | 98% | Yi et al., 2011 |
| _00003 | LacI family transcriptional regulator | | | | |
| _00004 | glycoside hydrolase GH51 family | alpha-L-arabinofuranosidase | PDB:2Y2W | 99% | |
| _00005 | sugar ABC transporter substrate-binding protein | COG1653 | | | |
| _00006 | sugar ABC transporter substrate-binding protein | COG1653 | | | |
| _00007 | sugar ABC transporter substrate-binding protein | COG1653 | | | |
| _00032 | glycoside hydrolase GH51 family | | | | |
| _00117 | glycoside hydrolase GH2 family | | | | |
| _00168 | glycoside hydrolase GH3 family* | | | | |
| _00202 | 1,4-alpha-glucan branching enzyme GlgB | | | | |
| _00220 | glycogen debranching enzyme GlgX | | | | |
| _00226 | glycoside hydrolase GH2 family | beta-galactosidase | AAL02052 | 96% | Hung et al., 2001 |
| _00227 | galactoside symporter | | | | |
| _00228 | glycoside hydrolase GH42 family | | | | |
| _00236 | glycoside hydrolase GH3 family | beta-D-glucosidase | ADY62498 | 96% | Jung et al., 2012 |
| _00237 | TetR family transcriptional regulator | | | | |
| _00238 | glycoside hydrolase GH30 family | beta-D-xylosidase | ABX45137 | 87% | Hyun et al., 2012 |
| _00239 | hypothetical protein | | | | |
| _00240 | glycoside hydrolase GH42 family | | | | |
| _00241 | MFS transporter | | | | |
| _00285 | NagC family transcriptional regulator | | | | |
| _00286 | xylulokinase XylB | | | | |
| _00287 | hypothetical protein | | | | |
| _00288 | hypothetical protein | | | | |
| _00289 | sugar ABC transporter permease | pseudogene | | | |
| _00290 | xylose isomerase XylA | | | | |
| _00299 | pentose ABC transporter permease | | | | |
| _00300 | pentose ABC transporter ATP-binding protein | | | | |
| _00301 | pentose ABC transporter substrate-binding protein | COG4213 | | | |
| | NagC family transcriptional regulator/sugar kinase | | | | |

| | | | | | |
|---|---|---|---|---|---|
| _00302<br><br>_00303<br>_00304 | pentose ABC transporter ATP-binding protein<br>NagC family transcriptional regulator/sugar kinase | | | | |
| _00357<br>_00358<br>_00359<br>_00360<br>_00361<br>_00362<br>_00363 | UDP-glucose 4-epimerase GalE<br>galactose-1-phosphate uridylyltransferase GalT<br>N-acetylhexosamine 1-phosphate kinase<br>glycoside hydrolase GH112 family<br>sugar ABC transporter permease<br>sugar ABC transporter permease<br>sugar ABC transporter substrate-binding protein | <br><br><br>lacto-N-biose phosphorylase<br><br><br>COG1653 | <br><br>PDB:4WH1<br>PDB:2ZUS | <br><br>99%<br>96% | <br><br>Nishimoto & Kitaoka, 2007<br>Hidaka et al., 2009 |
| _00427<br>_00428<br>_00429<br>_00430<br>_00431<br>_00432<br>_00433<br>_00434<br><br>_00435<br><br>_00436<br><br>_00437<br>_00438 | glycoside hydrolase GH85 family<br>glycoside hydrolase GH85 family<br>glycoside hydrolase GH85 family<br>LacI family transcriptional regulator<br>N-acylglucosamine 2-epimerase<br>glycoside hydrolase GH125 family<br>PfkB family sugar kinase<br>NagC family transcriptional regulator/sugar kinase<br>NagC family transcriptional regulator/sugar kinase<br>NagC family transcriptional regulator/sugar kinase<br>glucosamine-6-phosphate deaminase NagB<br>N-acetylglucosamine-6-phosphate deacetylase NagA | pseudogene?<br>pseudogene?<br>pseudogene? | | | |
| _00596 | glycoside hydrolase GH13 family | | | | |
| _00688 | glycoside hydrolase GH129 family | alpha-N-acetylgalactosaminidase | BAL14929 | 77% | Kiyohara et al., 2012 |
| _00701 | glycoside hydrolase GH20 family | | | | |
| _00719<br>_00720<br>_00721<br>_00722 | sugar ABC transporter permease<br>sugar ABC transporter permease<br>sugar ABC transporter ATP-binding protein<br>sugar ABC transporter substrate-binding protein | COG1172<br>COG1172<br>COG1129<br>COG1879 | | | Gene cluster associated with fructose metabolism (Fukuda et al., 2011) |
| _00805<br>_00806<br>_00807<br>_00808<br>_00809 | AraC family transcriptional regulator<br>glycoside hydrolase GH1 family<br>sugar ABC transporter permease<br>sugar ABC transporter permease<br>sugar ABC transporter substrate-binding protein | <br><br><br><br>COG1653 | | | |
| _00851 | lytic transglycosylase GH23 family* | | | | |
| _00887<br>_00888<br>_00889 | 4-alpha-glucanotransferase GH77 family<br>LacI family transcriptional regulator<br>glycoside hydrolase GH13 family | | | | |
| _00898<br>_00899 | MFS transporter<br>sucrose phosphorylase GH13 family | <br>sucrose phosphorylase/transglucosylase | <br>AAO84039 | <br>96% | <br>Kim et al., 2003 |

| | | | | | |
|---|---|---|---|---|---|
| _00900 | hypothetical protein | | | | |
| _00901 | LacI family transcriptional regulator | | | | |
| _00973 | glycoside hydrolase GH101 family* | endo-alpha-N-acetylgalactosaminidase | PDB:2ZXQ | 93% | Suzuki et al., 2009 |
| _01026 | glycoside hydrolase GH30 family* | | | | |
| _01027 | glycoside hydrolase GH43 family* | | | | |
| _01041 | glycoside hydrolase GH127 family | beta-L-arabinofuranosidase | PDB:3WKW | 95% | Ito et al., 2014 |
| _01042 | AraC family transcriptional regulator | | | | |
| _01043 | LacI family transcriptional regulator | | | | |
| _01044 | glycoside hydrolase GH27 family | | | | |
| _01106 | sugar ABC transporter substrate-binding protein | COG1653 | | | |
| _01107 | sugar ABC transporter permease | | | | |
| _01108 | sugar ABC transporter permease | | | | |
| _01109 | glycoside hydrolase GH42 family | beta-galactosidase | ADO51667 | 97% | O'Connell-Motherway et al., 2012 |
| _01110 | LacI family transcriptional regulator | | | | |
| _01111 | glycoside hydrolase GH53 family* | beta-1,4-endogalactanase | ADO51669 | 97% | O'Connell-Motherway et al., 2010 |
| _01178 | glycogen debranching enzyme GlgX | | | | |
| _01181 | 4-alpha-glucanotransferase GH77 family | | | | |
| _01222 | glycoside hydrolase GH13 family | pseudogene | | | |
| _01242 | LacI family transcriptional regulator | | | | |
| _01243 | glycoside hydrolase GH95 family | | | | |
| _01303 | lytic transglycosylase GH23 family | | | | |
| _01307 | glycoside hydrolase GH3 family | | | | |
| _01310 | glycoside hydrolase GH3 family | | | | |
| _01318 | sugar ABC transporter ATP-binding protein | COG3839 | | | |
| _01322 | PTS system N-acetylglucosamine-specific IIBC component | | | | |
| _01323 | PTS system N-acetylglucosamine-specific IIA component | | | | |
| _01324 | LacI family transcriptional regulator | | | | |
| _01332 | glycoside hydrolase GH36 family | | | | Gene cluster associated with melibiose and raffinose utilization (Anderson et al., 2013) |
| _01333 | NagC family transcriptional regulator/sugar kinase | | | | |
| | sugar ABC transporter substrate-binding protein | | | | |
| _01334 | sugar ABC transporter permease | COG1653 | | | |
| _01335 | sugar ABC transporter permease | | | | |
| _01336 | hypothetical protein | | | | |
| _01337 | glycoside hydrolase GH13 family | | | | |
| _01338 | | | | | |
| _01451 | glycoside hydrolase GH13 family | | | | |
| _01504 | glycoside hydrolase GH32 family | beta-(1-2)-fructofuranosidase | ADG63078 | 98% | Turroni et al., 2010 |
| _01505 | oligosaccharide:H+ symporter | | | | |
| _01506 | LacI family transcriptional regulator | | | | |

| | | | | | |
|---|---|---|---|---|---|
| _01552<br>_01553<br>_01554<br>_01555<br>_01556<br>_01557<br>_01558<br>_01559 | glycoside hydrolase<br>glycoside hydrolase GH3 family<br>glycoside hydrolase GH5 family<br>sugar ABC transporter permease<br>sugar ABC transporter permease<br>sugar ABC transporter substrate-binding protein<br>glycoside hydrolase GH38 family<br>glycoside hydrolase GH38 family | <br><br><br><br><br>COG1653 | | | |
| _01656<br>_01657<br>_01658<br>_01659<br>_01660<br>_01661<br>_01662<br>_01663<br>_01664 | 4-alpha-glucanotransferase GH77 family<br>TraX family protein<br>pullulanase GH13 family<br>sugar ABC transporter permease<br>sugar ABC transporter permease<br>sugar ABC transporter substrate-binding protein<br>glycoside hydrolase GH13 family<br>HAD superfamily hydrolase<br>LacI family transcriptional regulator | <br><br><br><br><br>COG2182 maltose-binding | | | |
| _01706<br>_01707<br>_01708 | glycoside hydrolase GH2 family<br>glycoside hydrolase GH2 family<br>LacI family transcriptional regulator | pseudogene<br>pseudogene | | | |
| _01718 | glycoside hydrolase GH3 family | alpha-L-arabinopyranosidase/beta-D-galactopyranosidase | ADT80794 | 99% | Lee et al., 2011 |
| _01856<br>_01857<br>_01858<br>_01859<br>_01860 | LacI family transcriptional regulator<br>sugar ABC transporter substrate-binding protein<br>sugar ABC transporter permease<br>sugar ABC transporter permease<br>glycoside hydrolase GH31 family | <br>COG1653 | | | |
| _01862<br>_01863<br>_01864<br>_01865 | sugar ABC transporter permease<br>sugar ABC transporter permease<br>LacI family transcriptional regulator<br>sugar ABC transporter substrate-binding protein | <br><br><br>COG1653 | | | |
| _01914<br>_01915<br>_01916<br>_01917<br>_01918<br>_01919<br>_01920 | glycoside hydrolase GH43 family<br>hypothetical protein<br>sugar ABC transporter permease<br>sugar ABC transporter permease<br>glycoside hydrolase GH13 family<br>sugar ABC transporter substrate-binding protein<br>LacI family transcriptional regulator | <br><br><br><br><br>COG1653 | | | |
| _01954<br>_01955<br>_01956 | glycoside hydrolase GH13 family<br>LacI family transcriptional regulator<br>sugar ABC transporter substrate-binding protein | <br><br>COG1653 | | | |

B. *Bifidobacterium pseudolongum* subsp. *globosum* AGR2145

| Locus tag G627DRAFT | Gene name | | | | |
|---|---|---|---|---|---|
| _00187<br>_00188<br>_00189 | cell surface protein*<br>glycoside hydrolase GH13 family*<br>pullulanase GH13 family* | | | | |
| _00212 | glycoside hydrolase GH13 family | | | | |
| _00421<br>_00422<br>_00423<br>_00424<br>_00425<br>_00426<br><br>_00427 | glycoside hydrolase GH13 family<br>glycoside hydrolase GH36 family<br>sugar ABC transporter permease<br>sugar ABC transporter permease<br>sugar ABC transporter substrate-binding protein<br>NagC family transcriptional regulator/sugar kinase<br>glycoside hydrolase GH36 family | COG1653 | | | Gene cluster associated with melibiose and raffinose utilization (Anderson et al., 2013) |
| _00436 | sugar ABC transporter ATP-binding protein | COG3839 | | | |
| _00442 | lytic transglycosylase GH23 family* | | | | |
| _00450<br>_00451<br>_00452<br>_00453 | Ribokinase<br>MFS transporter<br>inosine-uridine nucleoside N-ribohydrolase<br>LacI family transcriptional regulator | | | | |
| _00509 | glycoside hydrolase GH13 family* | | | | |
| _00515<br>_00516<br>_00517<br>_00518<br>_00519<br><br>_00520<br><br>_00521<br>_00522<br>_00523<br>_00524<br>_00525<br>_00526 | LacI family transcriptional regulator<br>hypothetical protein<br>pentose ABC transporter permease<br>pentose ABC transporter ATP-binding protein<br>pentose ABC transporter substrate-binding protein<br>NagC family transcriptional regulator/sugar kinase<br>pentose ABC transporter ATP-binding protein<br>hypothetical protein<br>glycoside hydrolase GH51 family<br>ribulokinase<br>L-ribulose-5-phosphate 4-epimerase AraD<br>L-arabinose isomerase AraA | | | | |
| _00534 | glycogen/starch/alpha-glucan phosphorylase | | | | |
| _00627<br>_00628<br>_00629 | LacI family transcriptional regulator<br>glycoside hydrolase GH2 family<br>galactoside symporter | | | | |
| _00675<br>_00676<br>_00677 | LacI family transcriptional regulator<br>oligosaccharide:H+ symporter<br>glycoside hydrolase GH32 family | beta-(1-2)-fructofuranosidase | ADG63078 | 75% | Turroni et al., 2010 |
| _00794<br>_00795 | glycoside hydrolase GH94 family<br>N-acylglucosamine 2-epimerase | | | | |

| | | | | | |
|---|---|---|---|---|---|
| _00796 | glycoside hydrolase GH36 family | | | | |
| _00797 | hypothetical protein | | | | |
| _00798 | hypothetical protein | | | | |
| _00799 | LacI family transcriptional regulator | | | | |
| _00800 | glycoside hydrolase GH2 family | | | | |
| _00801 | sugar ABC transporter permease | | | | |
| _00802 | sugar ABC transporter permease | | | | |
| _00803 | sugar ABC transporter substrate-binding protein | COG1653 | | | |
| _00804 | glycoside hydrolase GH1 family | beta-D-glucosidase/beta-D-fucosidase/transglycosylase | BAA19881 | 93% | Nunoura et al., 1996 |
| _00811 | MFS transporter | | | | |
| _00812 | addiction module antitoxin RelB/DinJ family | | | | |
| _00813 | PIN domain-containing protein | | | | |
| _00814 | sucrose phosphorylase GH13 family | sucrose phosphorylase/transglucosylase | AAO84039 | 84% | Kim et al., 2003 |
| _00815 | LacI family transcriptional regulator | | | | |
| _00846 | 4-alpha-glucanotransferase GH77 family | | | | |
| _00851 | glycogen debranching enzyme GlgX | | | | |
| _00852 | NagC family transcriptional regulator/sugar kinase | | | | |
| _00898 | alpha-D-glucose phosphate-specific phosphoglucomutase | | | | |
| _00899 | PTS system mannose-specific IIABC component | | | | |
| _01071 | glycoside hydrolase GH13 family | | | | |
| _01072 | sugar ABC transporter substrate-binding protein | COG1653 | | | |
| _01073 | LacI family transcriptional regulator | | | | |
| _01074 | sugar ABC transporter permease | | | | |
| _01075 | sugar ABC transporter permease | | | | |
| _01076 | hypothetical protein | | | | |
| _01077 | 4-alpha-glucanotransferase GH77 family | | | | |
| _01078 | glycoside hydrolase GH13 family | | | | |
| _01079 | sugar ABC transporter substrate-binding protein | COG2182 maltose-binding | | | |
| _01080 | sugar ABC transporter permease | | | | |
| _01081 | sugar ABC transporter permease | | | | |
| _01082 | pullulanase GH13 family | | | | |
| _01083 | HAD superfamily hydrolase | | | | |
| _01084 | TraX family protein | | | | |
| _01085 | LacI family transcriptional regulator | | | | |
| _01091 | glycoside hydrolase GH3 family | | | | |
| _01092 | N-acetylglucosamine-6-phosphate deacetylase NagA | | | | |
| | glucosamine-6-phosphate deaminase NagB | | | | |
| _01093 | NagC family transcriptional regulator/sugar kinase | | | | |
| _01094 | NagC family transcriptional regulator/sugar kinase | | | | |
| | hypothetical protein | | | | |
| _01095 | sugar ABC transporter substrate-binding protein | | | | |
| | sugar ABC transporter permease | | | | |

| | | | | | |
|---|---|---|---|---|---|
| _01096<br>_01097<br>_01098<br>_01099<br>_01100<br>_01101<br>_01102<br>_01103<br>_01104<br>_01105<br>_01106<br>_01107<br>_01108<br>_01109<br>_01110<br>_01111<br>_01112<br>_01113<br>_01114<br>_01115<br>_01116<br>_01117<br>_01118<br>_01119<br>_01120 | sugar ABC transporter permease<br>lacto-N-biose phosphorylase GH112 family<br>N-acetylhexosamine 1-phosphate kinase<br>galactose-1-phosphate uridylyltransferase GalT<br>flavodoxin family protein<br>tRNA_Ala<br>LacI family transcriptional regulator<br>sugar ABC transporter substrate-binding protein<br>sugar ABC transporter permease<br>sugar ABC transporter permease<br>glycoside hydrolase GH31 family<br>GtrA family protein<br>hypothetical protein<br>hypothetical protein<br>sugar ABC transporter permease<br>sugar ABC transporter permease<br>sugar ABC transporter substrate-binding protein<br>alpha-L-fucosidase GH29 family<br>glycoside hydrolase GH3 family<br>glycoside hydrolase GH31 family<br>glycoside hydrolase GH42 family<br>NagC family transcriptional regulator/sugar kinase | COG1653<br>COG1653<br>COG1653 | PDB:2ZUS<br>PDB:4WH1 | 77%<br>65% | Hidaka et al., 2009<br>Nishimoto & Kitaoka, 2007 |
| _01131 | glycoside hydrolase GH36 family | | | | |
| _01171<br>_01172<br>_01174<br>_01175<br>_01176<br>_01177<br>_01178<br>_01179<br>_01180<br>_01181<br>_01182<br>_01183 | xylose isomerase XylA<br>glycoside hydrolase GH43 family<br>LacI family transcriptional regulator<br>sugar ABC transporter substrate-binding protein<br>sugar ABC transporter permease<br>sugar ABC transporter permease<br>glycoside hydrolase GH43 family<br>acetylxylan esterase<br>esterase<br>glycoside hydrolase GH43 family<br>xylulokinase XylB<br>NagC family transcriptional regulator/sugar kinase | COG1653 | PDB:3ZKK | 98% | Gene cluster associated with XOS metabolism (Andersen et al., 2013)<br>Ejby et al., 2013 |
| _01240 | sugar ABC transporter substrate-binding protein | COG1653 | | | |
| _01249 | glycoside hydrolase GH30 family | | | | |
| _01275 | 1,4-alpha-glucan branching enzyme GlgB | | | | |
| _01302 | glycoside hydrolase GH3 family* | | | | |
| _01669 | glycoside hydrolase GH13 family* | Incomplete at C-terminal | | | |
| _01689<br>_01690 | LacI family transcriptional regulator<br>sugar ABC transporter substrate-binding protein | COG1653 | | | |

| | | | | | |
|---|---|---|---|---|---|
| _01691<br>_01692<br>_01693 | sugar ABC transporter permease<br>sugar ABC transporter permease<br>glycoside hydrolase GH31 family | | | | |
| _01702 | glycoside hydrolase GH13 family* | Incomplete at C-terminal | | | |
| _01708 | glycoside hydrolase GH13 family* | Incomplete at C-terminal | | | |

## C. *Bifidobacterium choerinum* AGR2158

| Locus tag G606DRAFT | Gene name | | | | |
|---|---|---|---|---|---|
| _00044<br>_00045<br>_00046<br>_00047<br>_00048<br>_00049<br>_00050<br>_00051<br>_00052<br>_00053<br>_00054<br>_00055<br>_00056<br>_00057<br>_00058 | glycoside hydrolase GH13 family<br>sugar ABC transporter substrate-binding protein<br>LacI family transcriptional regulator<br>sugar ABC transporter permease<br>sugar ABC transporter permease<br>hypothetical protein<br>4-alpha-glucanotransferase GH77 family<br>glycoside hydrolase GH13 family<br>sugar ABC transporter substrate-binding protein<br>sugar ABC transporter permease<br>sugar ABC transporter permease<br>pullulanase GH13 family<br>HAD superfamily hydrolase<br>TraX family protein<br>LacI family transcriptional regulator | <br>COG1653<br><br><br><br><br><br><br>COG2182 maltose-binding | | | |
| _00075 | glycoside hydrolase GH13 family* | Incomplete at C-terminal | | | |
| _00078 | glycoside hydrolase GH13 family* | Incomplete at C-terminal | | | |
| _00318 | sugar ABC transporter substrate-binding protein | COG1653 | | | |
| _00353<br><br>_00354<br>_00355<br>_00356<br>_00357<br>_00358<br>_00359<br>_00360<br>_00361<br>_00362<br>_00363<br>_00364 | NagC family transcriptional regulator/sugar kinase<br>xylulokinase XylB<br>glycoside hydrolase GH43 family<br>esterase<br>acetylxylan esterase<br>glycoside hydrolase GH43 family<br>sugar ABC transporter permease<br>sugar ABC transporter permease<br>sugar ABC transporter substrate-binding protein<br>LacI family transcriptional regulator<br>glycoside hydrolase GH43 family<br>xylose isomerase XylA | <br><br><br><br><br><br><br><br><br>COG1653 | <br><br><br><br><br><br><br><br><br>PDB:3ZKK | <br><br><br><br><br><br><br><br><br>98% | Gene cluster associated with<br>XOS metabolism (Andersen et<br>al., 2013)<br><br><br><br><br><br><br>Ejby et al., 2013 |
| _00383 | glycoside hydrolase GH94 family | | | | |
| _00444 | alpha-D-glucose phosphate-specific phosphoglucomutase | | | | |

| | | | | | |
|---|---|---|---|---|---|
| _00490<br><br>_00491<br>_00492 | NagC family transcriptional regulator/sugar kinase<br>hypothetical protein<br>glycogen debranching enzyme GlgX | | | | |
| _00499 | 4-alpha-glucanotransferase GH77 family | | | | |
| _00722<br>_00723<br>_00724<br>_00725<br>_00726<br>_00727<br>_00728<br>_00729<br>_00730<br>_00731 | N-acylglucosamine 2-epimerase<br>glycoside hydrolase GH36 family<br>hypothetical protein<br>hypothetical protein<br>LacI family transcriptional regulator<br>glycoside hydrolase GH2 family<br>sugar ABC transporter permease<br>sugar ABC transporter permease<br>sugar ABC transporter substrate-binding protein<br>glycoside hydrolase GH1 family | COG1653<br>beta-D-glucosidase/beta-D-fucosidase/transglycosylase | BAA19881 | 89% | Nunoura et al., 1996 |
| _00749<br>_00750<br><br>_00751 | MFS transporter<br>sucrose phosphorylase GH13 family<br>LacI family transcriptional regulator | sucrose phosphorylase/transglucosylase | AAO84039 | 85% | Kim et al., 2003 |
| _00811 | glycogen/starch/alpha-glucan phosphorylase | | | | |
| _00820<br>_00821<br>_00822<br>_00823<br>_00824<br>_00825<br>_00826<br>_00827<br>_00828 | L-arabinose isomerase AraA<br>L-ribulose-5-phosphate 4-epimerase AraD<br>ribulokinase<br>MFS transporter<br>pentose ABC transporter permease<br>LacI family transcriptional regulator<br>LacI family transcriptional regulator<br>glycoside hydrolase GH42 family<br>galactoside symporter | pseudogene | | | |
| _00908 | lytic transglycosylase GH23 family* | | | | |
| _00914 | sugar ABC transporter ATP-binding protein | COG3839 | | | |
| _00925<br>_00926<br><br>_00927<br>_00928<br>_00929<br>_00930<br>_00931 | glycoside hydrolase GH36 family<br>NagC family transcriptional regulator/sugar kinase<br>sugar ABC transporter substrate-binding protein<br>sugar ABC transporter permease<br>sugar ABC transporter permease<br>glycoside hydrolase GH36 family<br>glycoside hydrolase GH13 family | COG1653 | | | Gene cluster associated with melibiose and raffinose utilization (Anderson et al., 2013) |
| _00966 | 1,4-alpha-glucan branching enzyme GlgB | | | | |
| _00995 | glycoside hydrolase GH3 family* | | | | |
| _01152 | NagC family transcriptional regulator/sugar kinase<br>PfkB family carbohydrate kinase | | | | |

| | | | | | |
|---|---|---|---|---|---|
| _01153 | | | | | |
| _01217<br>_01218<br>_01219 | glycoside hydrolase GH32 family<br>oligosaccharide:H+ symporter<br>LacI family transcriptional regulator | beta-(1-2)-fructofuranosidase | ADG63078 | 72% | Turroni et al., 2010 |
| _01269 | glycoside hydrolase GH5 family | | | | |
| _01584<br>_01585<br>_01586 | glycoside hydrolase GH13 family*<br>cell surface protein*<br>pullulanase GH13 family* | | | | |
| _01629 | glycoside hydrolase GH13 family | | | | |
| _01653 | glycoside hydrolase GH30 family | | | | |
| _01661 | glycoside hydrolase GH13 family* | Incomplete at C-terminal | | | |
| _01670 | glycoside hydrolase GH25 family* | | | | |
| _01686 | glycoside hydrolase GH36 family | | | | |
| _01694<br>_01695<br>_01696<br><br>_01697<br>_01698<br>_01699<br>_01700<br>_01701<br>_01702<br>_01703 | esterase<br>TetR family transcriptional regulator<br>NagC family transcriptional regulator/sugar kinase<br>glycoside hydrolase GH42 family<br>hypothetical protein<br>glycoside hydrolase GH31 family<br>sugar ABC transporter permease<br>sugar ABC transporter permease<br>sugar ABC transporter substrate-binding protein<br>LacI family transcriptional regulator | <br><br><br><br>pseudogene<br><br><br><br><br>COG1653 | | | |
| _01723 | glycoside hydrolase GH3 family | | | | |

* proteins with signal peptide sequences and predicted to be secreted or cell surface associated.

† Enzymes shown are homologous to those that have been characterized in other *Bifidobacterium* species.

**Table S3**. Scaffold location and locus tag numbers of exopolysaccharide (EPS) gene clusters in the AGR2137, AGR2145 and AGR2158 genomes.

| **AGR2158** | **AGR2145** | **AGR2137** |
|---|---|---|
| Eps3<br>G606DRAFT_1364-<br>G606DRAFT_1404<br>(G606DRAFT_scaffold00004.4) | Eps3<br>G627DRAFT_01479-<br>G627DRAFT_01524<br>(G627DRAFT_scaffold00013.13) | G629DRAFT_01890-<br>G629DRAFT_01902<br>(G629DRAFT_scaffold00032.32) |
| Eps4<br>G606DRAFT_1663-<br>G606DRAFT_1676<br>(G606DRAFT_scaffold00007.7) | Eps4<br>G627DRAFT_01141-<br>G627DRAFT_01152<br>(G627DRAFT_scaffold00008.8) | G629DRAFT_01905-<br>G629DRAFT_01910<br>(G629DRAFT_scaffold00033.33) |
| | | G629DRAFT_01937-<br>G629DRAFT_01941<br>(G629DRAFT_scaffold00037.37) |
| | | G629DRAFT_01944-<br>G629DRAFT_01946<br>(G629DRAFT_scaffold00038.38) |

Fig. S1

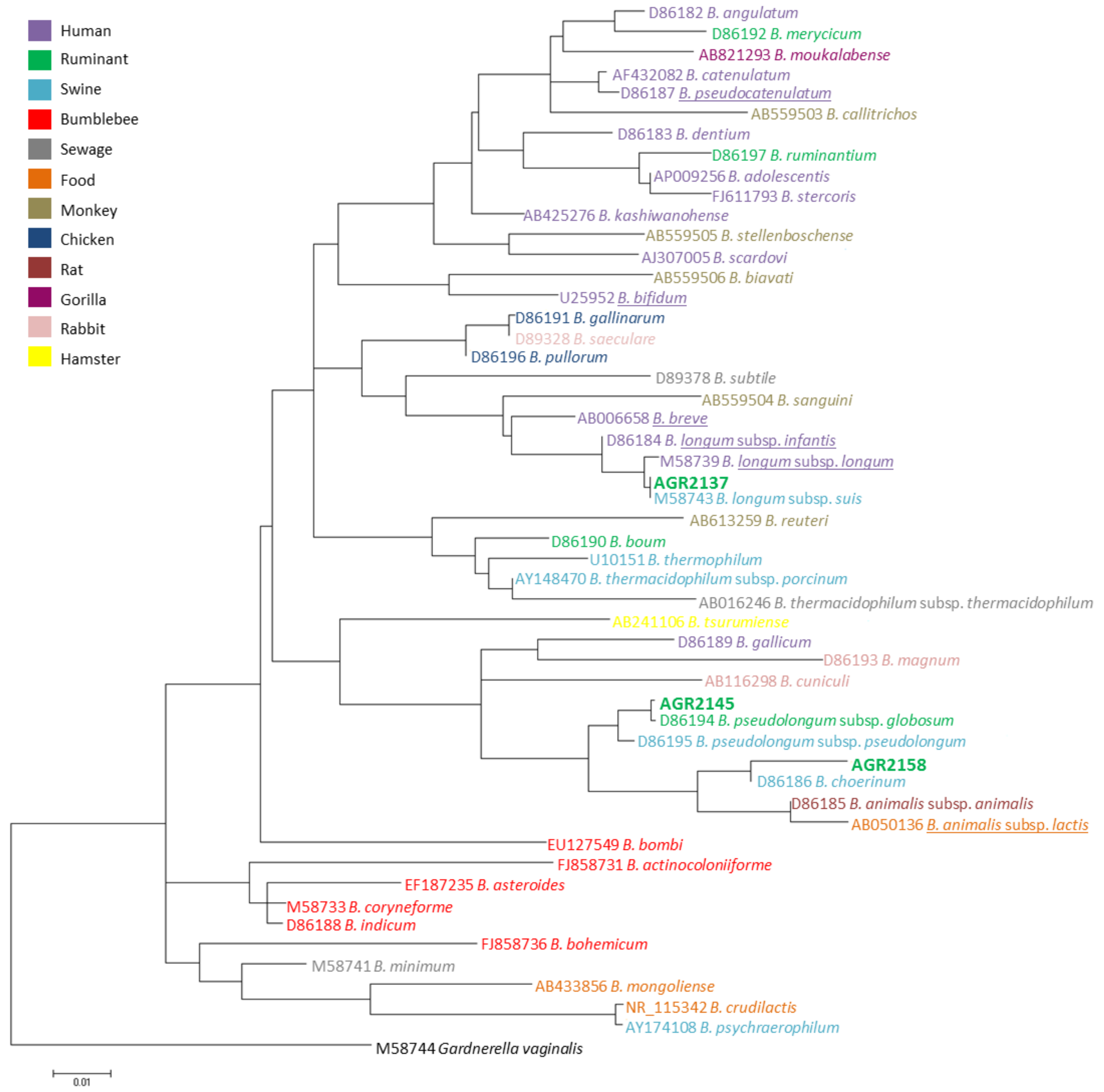

Human
Ruminant
Swine
Bumblebee
Sewage
Food
Monkey
Chicken
Rat
Gorilla
Rabbit
Hamster
D86182 B. angulatum
D86192 B. merycicum
AB821293 B. moukalabense
AF432082 B. catenulatum
D86187 B. pseudocatenulatum
AB559503 B. callitrichos
D86183 B. dentium
D86197 B. ruminantium
AP009256 B. adolescentis
FJ611793 B. stercoris
AB425276 B. kashiwanohense
AB559505 B. stellenboschense
AJ307005 B. scardovi
AB559506 B. biavati
U25952 B. bifidum
D86191 B. gallinarum
D89328 B. saeculare
D86196 B. pullorum
D89378 B. subtile
AB559504 B. sanguini
AB006658 B. breve
D86184 B. longum subsp. infantis
M58739 B. longum subsp. longum
AGR2137
M58743 B. longum subsp. suis
AB613259 B. reuteri
D86190 B. boum
U10151 B. thermophilum
AY148470 B. thermacidophilum subsp. porcinum
AB016246 B. thermacidophilum subsp. thermacidophilum
AB241106 B. tsurumiense
D86189 B. gallicum
D86193 B. magnum
AB116298 B. cuniculi
AGR2145
D86194 B. pseudolongum subsp. globosum
D86195 B. pseudolongum subsp. pseudolongum
AGR2158
D86186 B. choerinum
D86185 B. animalis subsp. animalis
AB050136 B. animalis subsp. lactis
EU127549 B. bombi
FJ858731 B. actinocoloniiforme
EF187235 B. asteroides
M58733 B. coryneforme
D86188 B. indicum
FJ858736 B. bohemicum
M58741 B. minimum
AB433856 B. mongoliense
NR_115342 B. crudilactis
AY174108 B. psychraerophilum
M58744 Gardnerella vaginalis
0.01

Fig. S2

(a) (b)

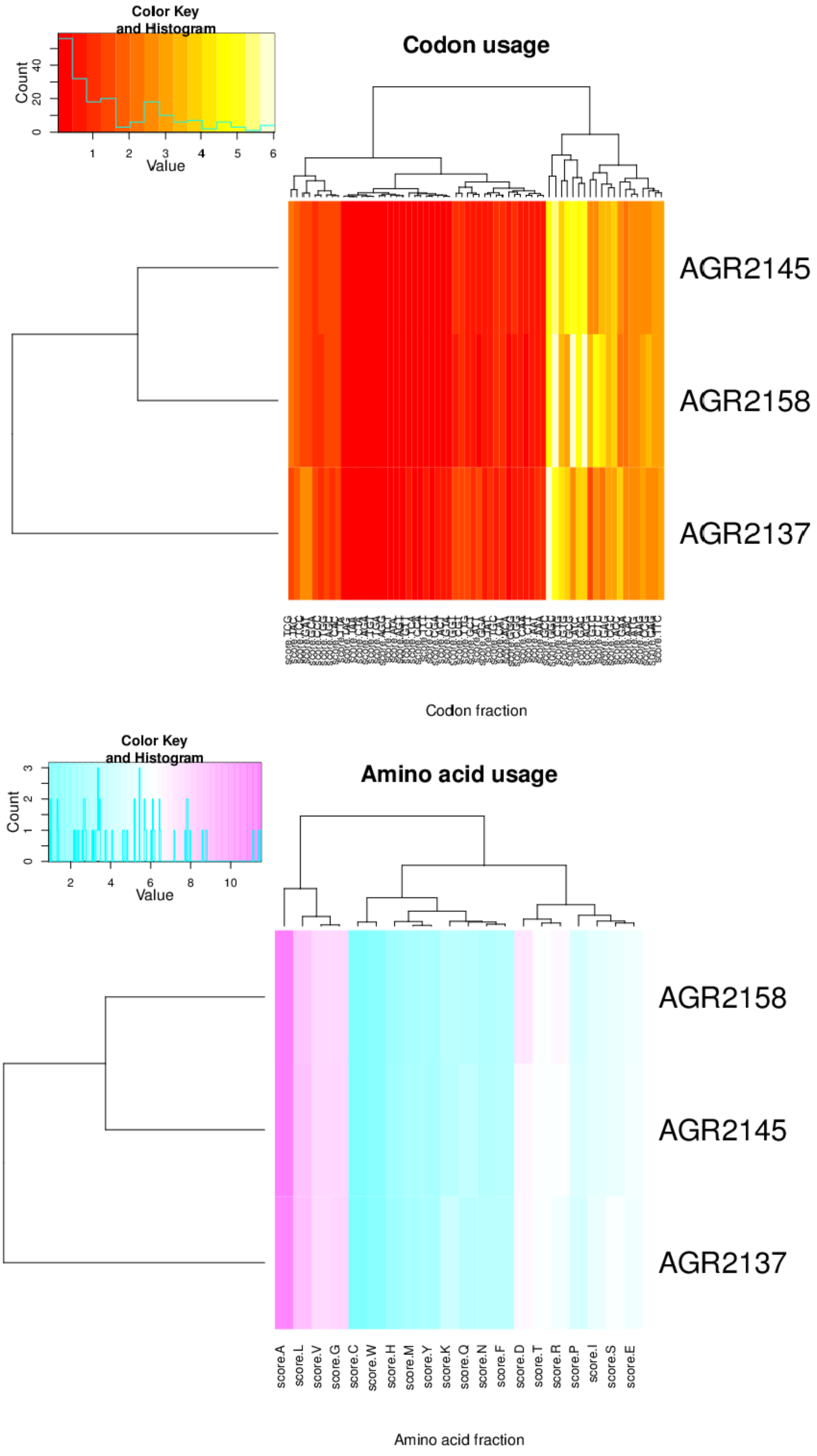

Color Key
and Histogram
Count
Value
Codon usage
AGR2145
AGR2158
AGR2137
Codon fraction
Color Key
and Histogram
Count
Value
Amino acid usage
AGR2158
AGR2145
AGR2137
score.A
score.L
score.V
score.G
score.C
score.W
score.H
score.M
score.Y
score.K
score.Q
score.N
score.F
score.D
score.T
score.R
score.P
score.I
score.S
score.E
Amino acid fraction

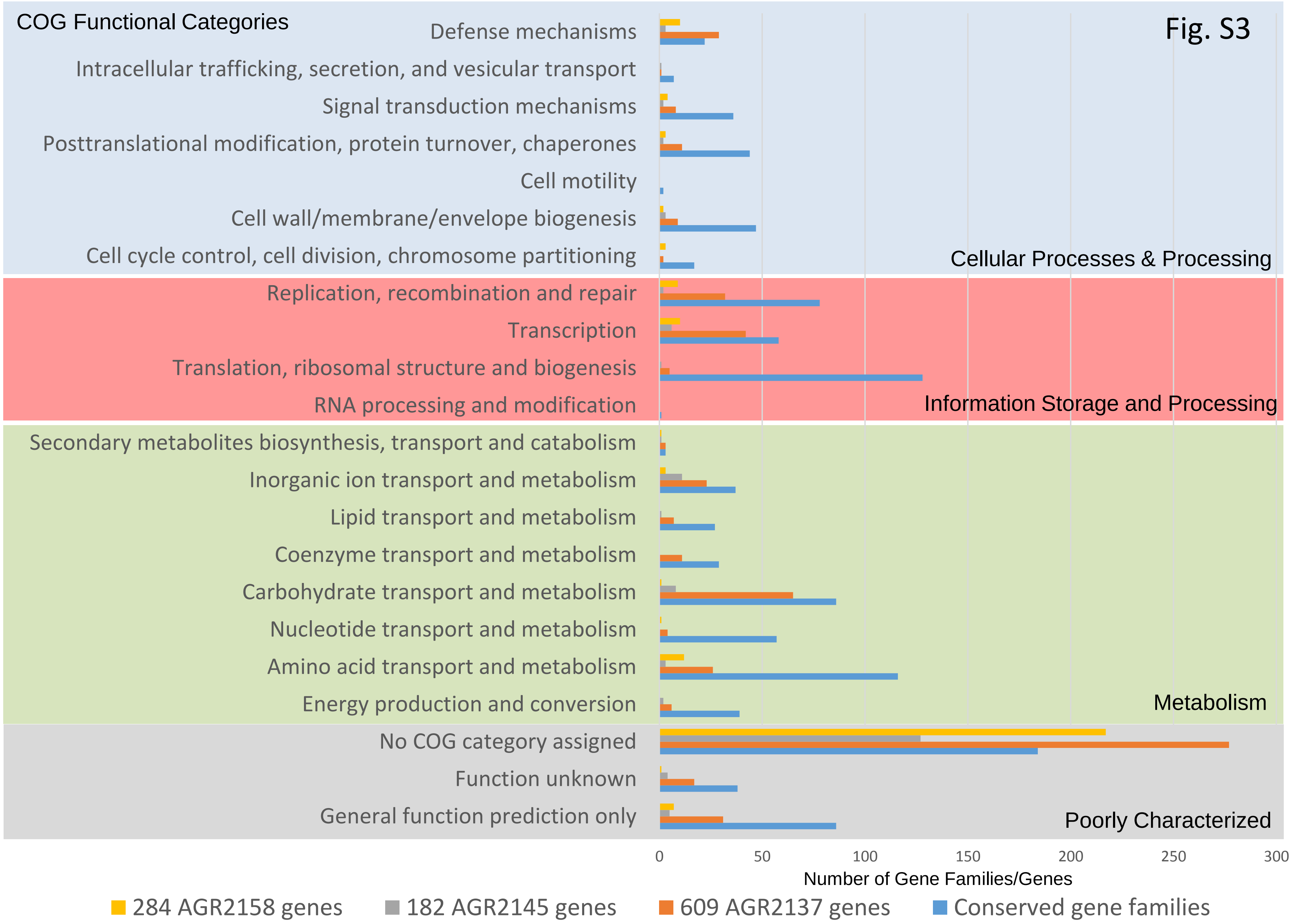

Fig. S3
COG Functional Categories
Defense mechanisms
Intracellular trafficking, secretion, and vesicular transport
Signal transduction mechanisms
Posttranslational modification, protein turnover, chaperones
Cell motility
Cell wall/membrane/envelope biogenesis
Cell cycle control, cell division, chromosome partitioning
Cellular Processes & Processing
Replication, recombination and repair
Transcription
Translation, ribosomal structure and biogenesis
RNA processing and modification
Information Storage and Processing
Secondary metabolites biosynthesis, transport and catabolism
Inorganic ion transport and metabolism
Lipid transport and metabolism
Coenzyme transport and metabolism
Carbohydrate transport and metabolism
Nucleotide transport and metabolism
Amino acid transport and metabolism
Energy production and conversion
Metabolism
No COG category assigned
Function unknown
General function prediction only
Poorly Characterized
0
50
100
150
200
250
300
Number of Gene Families/Genes
284 AGR2158 genes
182 AGR2145 genes
609 AGR2137 genes
Conserved gene families

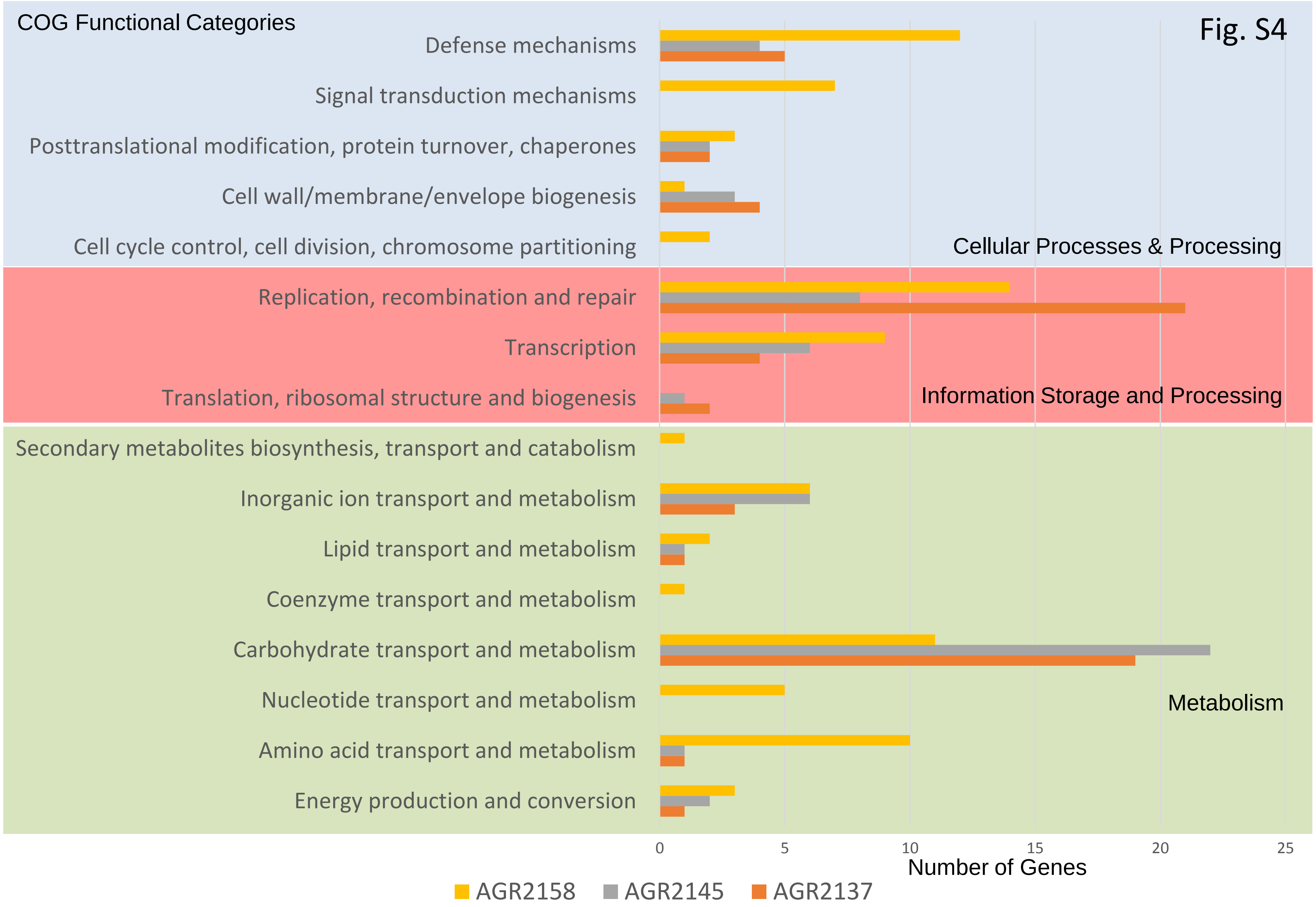

Fig. S4
COG Functional Categories
Defense mechanisms
Signal transduction mechanisms
Posttranslational modification, protein turnover, chaperones
Cell wall/membrane/envelope biogenesis
Cell cycle control, cell division, chromosome partitioning
Cellular Processes & Processing
Replication, recombination and repair
Transcription
Translation, ribosomal structure and biogenesis
Information Storage and Processing
Secondary metabolites biosynthesis, transport and catabolism
Inorganic ion transport and metabolism
Lipid transport and metabolism
Coenzyme transport and metabolism
Carbohydrate transport and metabolism
Nucleotide transport and metabolism
Amino acid transport and metabolism
Energy production and conversion
Metabolism
0
5
10
15
20
25
Number of Genes
AGR2158
AGR2145
AGR2137

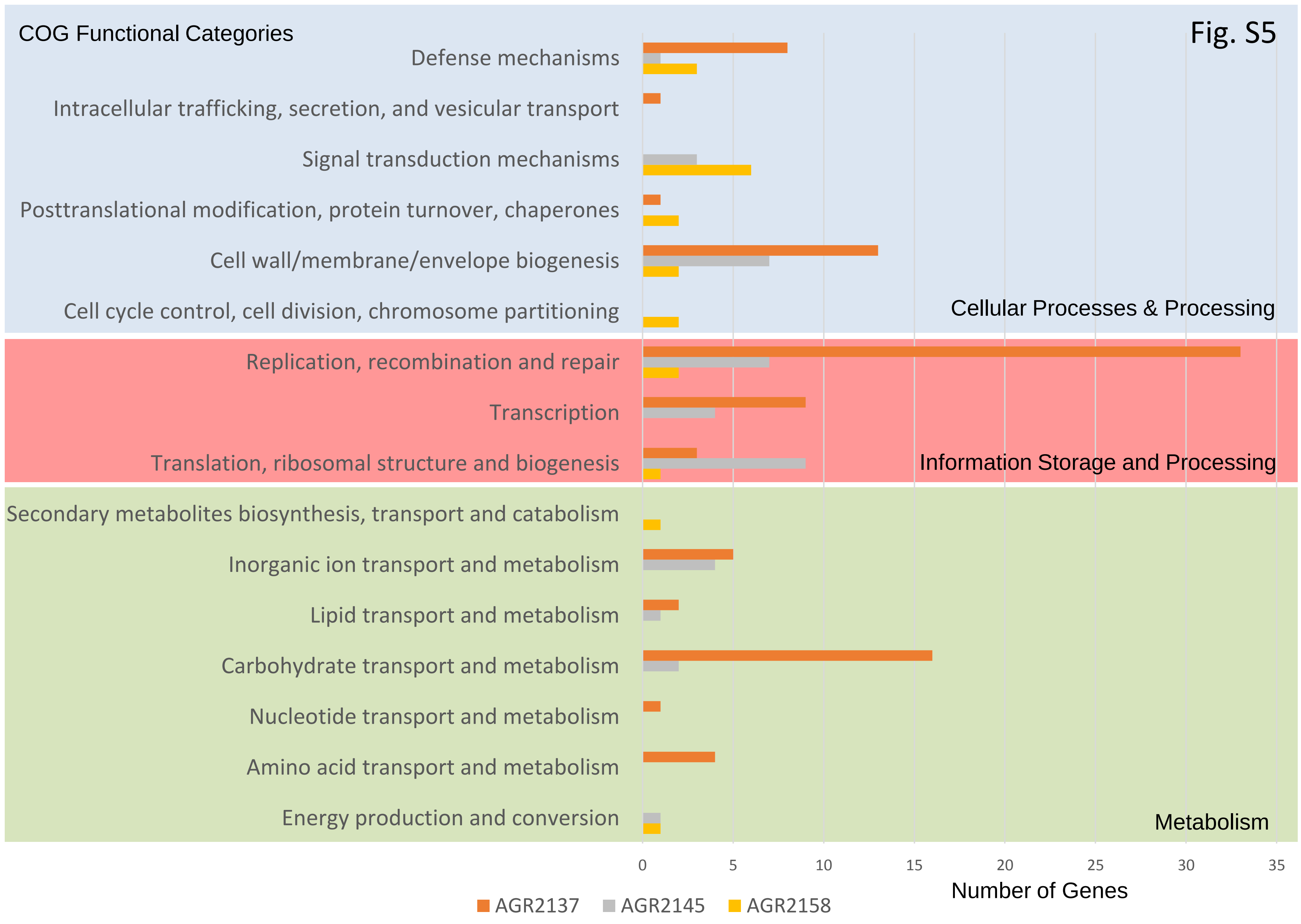

COG Functional Categories
Fig. S5
Defense mechanisms
Intracellular trafficking, secretion, and vesicular transport
Signal transduction mechanisms
Posttranslational modification, protein turnover, chaperones
Cell wall/membrane/envelope biogenesis
Cell cycle control, cell division, chromosome partitioning
Cellular Processes & Processing
Replication, recombination and repair
Transcription
Translation, ribosomal structure and biogenesis
Information Storage and Processing
Secondary metabolites biosynthesis, transport and catabolism
Inorganic ion transport and metabolism
Lipid transport and metabolism
Carbohydrate transport and metabolism
Nucleotide transport and metabolism
Amino acid transport and metabolism
Energy production and conversion
Metabolism
0
5
10
15
20
25
30
35
Number of Genes
AGR2137
AGR2145
AGR2158

Fig. S6

(a)

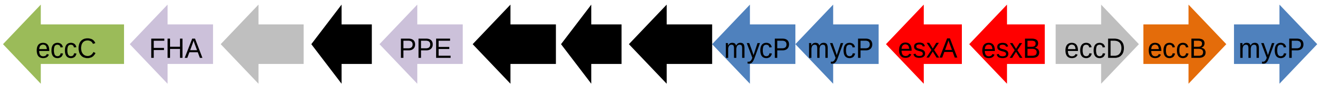


(b)

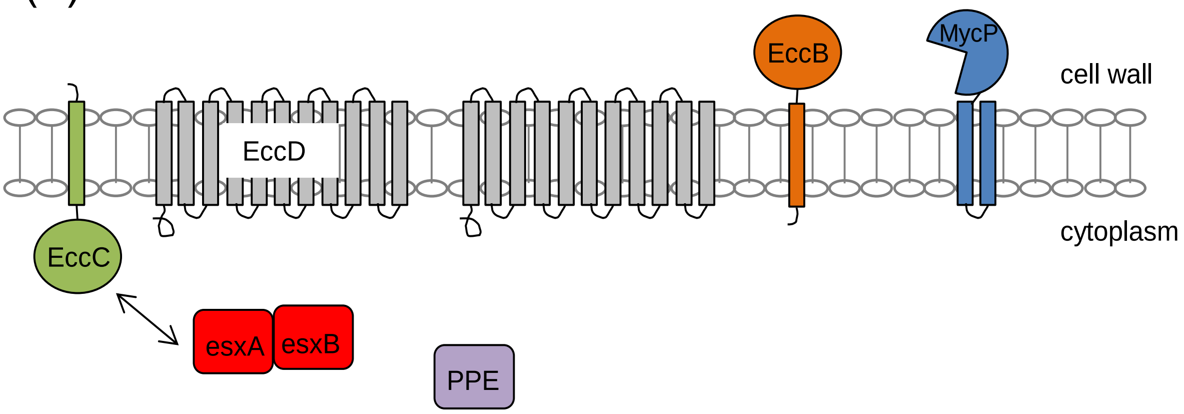


(c)

| Locus_tag | Description | Gene name | Bd1 locus_tag |
|---|---|---|---|
| G606Draft_1307 | FtsK/SpoIIIE-like transmembrane protein [1TM] | eccC | BDP_1565 |
| G606Draft_1308 | FHA-domain containing protein | | BDP_1566 |
| G606Draft_1309 | Transmembrane protein [11 TMs] | | BDP_1567 |
| G606Draft_1310 | Unknown | | BDP_1568 |
| G606Draft_1311 | PPE family protein | | BDP_1569 |
| G606Draft_1312 | Unknown | | BDP_1570 |
| G606Draft_1313 | Unknown | | BDP_1571 |
| G606Draft_1314 | Unknown | | BDP_1572 |
| G606Draft_1315 | Subtilisin-like serine protease (mycosin) [1 SP, 1 TM) | mycP | BDP_1573 |
| G606Draft_1316 | Subtilisin-like serine protease (mycosin) [1 SP, 1 TM) | mycP | BDP_1574 |
| G606Draft_1317 | WXG100 family secretion target | esxA | BDP_1575 |
| G606Draft_1318 | WXG100 family secretion target | esxB | BDP_1576 |
| G606Draft_1319 | Transmembrane protein [11 TMs] | eccD | BDP_1577 |
| G606Draft_1320 | Transmembrane protein [1 TMs] | eccB | BDP_1578 |
| G606Draft_1321 | Subtilisin-like serine protease (mycosin) [1 SP, 1 TM) | mycP | BDP_1579 |

TM transmembrane domain, SP Signal peptide sequence

Fig. S7

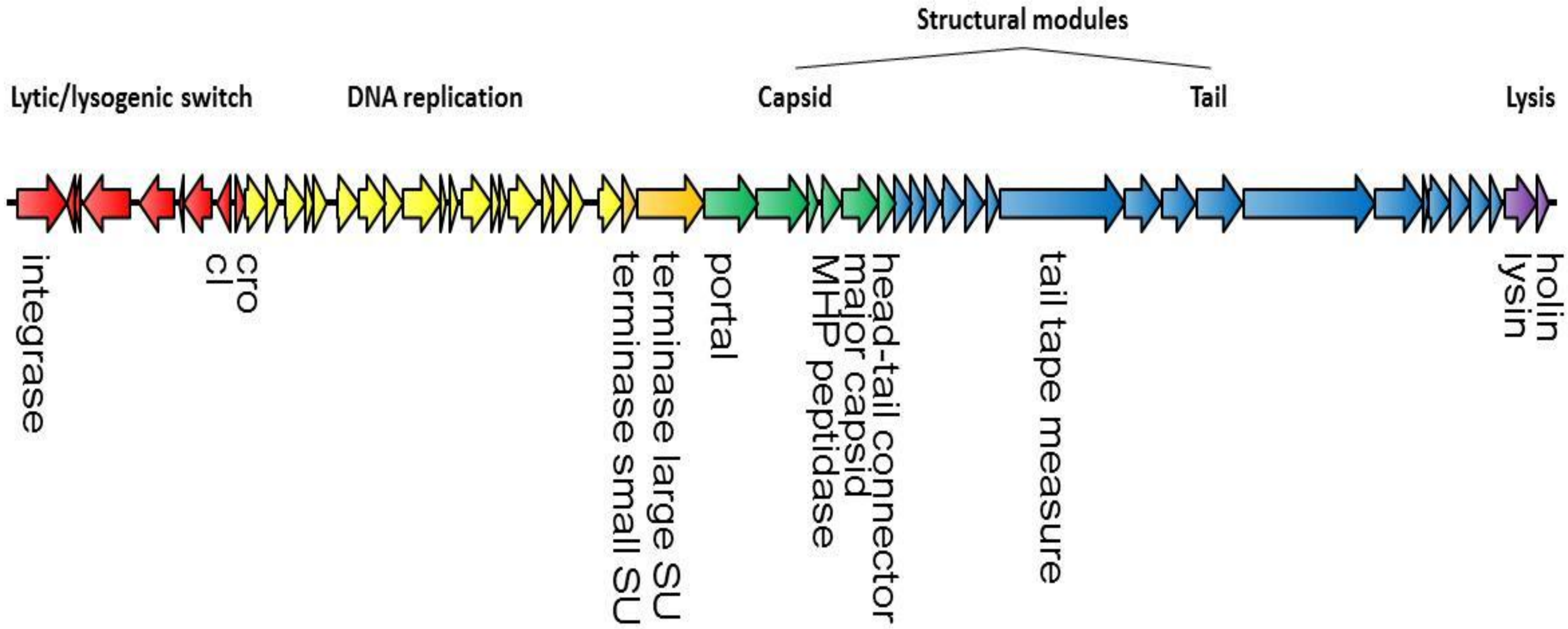
Structural modules
Lytic/lysogenic switch
DNA replication
Capsid
Tail
Lysis
integrase
cI
cro
terminase small SU
terminase large SU
portal
MHP peptidase
major capsid
head-tail connector
tail tape measure
lysin
holin

Fig. S8

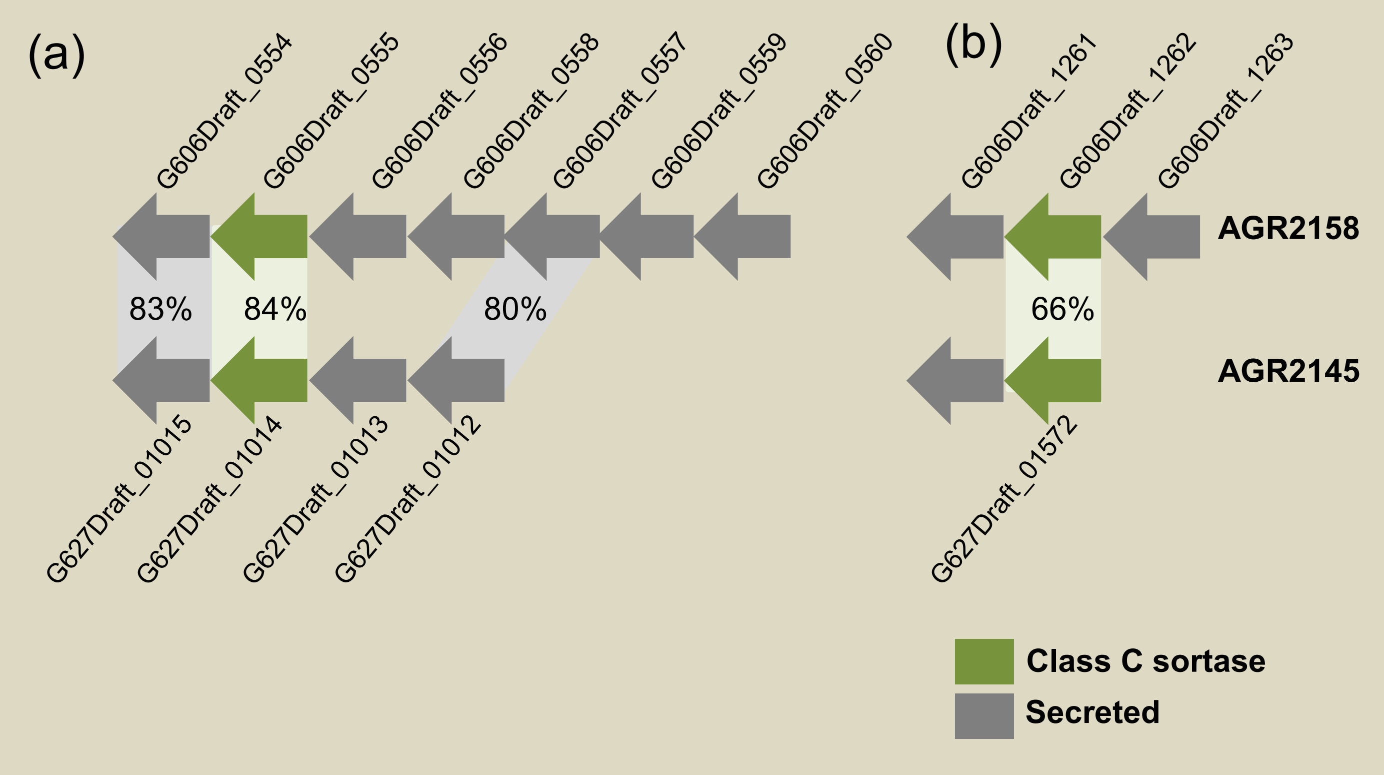
(a)
G606Draft_0554
G606Draft_0555
G606Draft_0556
G606Draft_0558
G606Draft_0557
G606Draft_0559
G606Draft_0560
83%
84%
80%
G627Draft_01015
G627Draft_01014
G627Draft_01013
G627Draft_01012
(b)
G606Draft_1261
G606Draft_1262
G606Draft_1263
AGR2158
66%
AGR2145
G627Draft_01572
Class C sortase
Secreted

## Supplementary References


Andersen, J.M., et al. Transcriptional analysis of oligosaccharide utilization by *Bifidobacterium lactis* Bl-04. *BMC Genomics* **14,** 312 (2013).

Di Gioia, D., Aloisio, I., Mazzola, G. & Biavati, B. Bifidobacteria: their impact on gut microbiota composition and their applications as probiotics in infants. *Appl. Microbiol.* Biotechnol. **98**, 563-577 (2014).

Ejby, M. et al. Structural basis for arabinoxylo-oligosaccharide capture by the probiotic *Bifidobacterium animalis* subsp. *lactis* Bl-04. *Mol. Microbiol.* **90,** 1100-1112 (2013).

Fukuda, S., et al. Bifidobacteria can protect from enteropathogenic infection through production of acetate. *Nature* **469,** 543-547 (2011).

Hidaka, M., et al. The crystal structure of galacto-N-biose/lacto-N-biose I phosphorylase. *J. Biol. Chem.* **284,** 7273-7283 (2009).

Hung, M-N., Xia, Z., Hu, N-T. & Lee, B.H. Molecular and biochemical analysis of two β-galactosidases from *Bifidobacterium infantis* HL96. *Appl. Environ. Microbiol.* **67,** 4256-4263 (2001).

Hyun, Y-J., Kim, B. & Kim, D.H. Cloning and characterization of ginsenoside Ra1-hydrolyzing beta-D-xylosidase from *Bifidobacterium breve* K-110. *J. Microbiol. Biotechnol.* **22,** 535-540 (2012).

Ito, T., et al. Crystal structure of glycoside hydrolase family 127 β-L-arabinofuranosidase from *Bifidobacterium longum*. *Biochem. Biophys. Res. Comm.* **447,** 32-37 (2014).

Jung, I-H., Lee, J.H., Hyun, Y-J. & Kim, D-H. Metabolism of ginsenoside Rb1 by human intestinal microflora and cloning of its metabolizing β-D-glucosidase from *Bifidobacterium longum* H-1. *Biol. Pharm. Bull.* **35,** 573-581 (2012).

Kim, M., et al. Cloning and expression of sucrose phosphorylase gene from *Bifidobacterium longum* in *E. coli* and characterization of the recombinant enzyme. *Biotechnol. Lett.* **25,** 1211-1217 (2003).

Kiyohara, M., et al. α-N-acetylgalactosaminidase from infant-associated bifidobacteria belonging to novel glycoside hydrolase family 129 is implicated in alternative mucin degradation pathway. *J. Biol. Chem.* **287,** 693-700 (2012).

Lee, J.H., Hyun, Y-J. & Kim, D-H. Cloning and characterization of α-L-arabinofuranosidase and bifunctional α-L-arabinopyranosidase/β-D-galactopyranosidase from *Bifidobacterium longum* H-1. *J. Appl. Microbiol.* **111,** 1097-1107 (2011).

Lugli et al. Investigation of the evolutionary development of the genus *Bifidobacterium* by comparative genomics. *Appl. Environ. Microbiol.* 80, 6383-6394 (2014).

Nishimoto, M. & Kitaoka, M. Identification of N-acetylhexosamine 1-kinase in the complete lacto-N-biose I/galacto-N-biose metabolic pathway in *Bifidobacterium longum*. *Appl. Environ. Microbiol.* **73,** 6444-6449 (2007).

Nunoura, N., et al. Cloning and nucleotide sequence of the β-D-glucosidase gene from *Bifidobacterium breve* clb and expression of β-D-glucosidase activity in *Escherichia coli*. *Biosci. Biotech. Biochem.* **60,** 2011-2018 (2011)..

O'Connell Motherway, M., Fitzgerald, G. F. & van Sinderen, D. Metabolism of a plant derived galactose-containing polysaccharide by *Bifidobacterium breve* UCC2003. *Microb. Biotechnol.* **4,** 403-416 (2010).

O'Connell Motherway, M., Kinsella, M., Fitzgerald, G. F. & van Sinderen, D. Transcriptional and functional characterization of genetic elements involved in galacto-oligosaccharide utilization by *Bifidobacterium breve* UCC2003. *Microb. Biotechnol.* **6,** 67-79 (2012).

Suzuki, R., et al. Crystallographic and mutational analyses of substrate recognition of endo-α-acetylgalactosaminidase from *Bifidobacterium longum*. *J. Biochem.* **146,** 389-398 (2009).

Tamura, K., Stecher, G., Peterson, D., Filipski, A. & Kumar, S. MEGA6: Molecular Evolutionary Genetics Analysis version 6.0. *Mol. Biol. Evol.* **30**, 2725-2729 (2013).

Turroni, F., et al. Characterization of the serpin-encoding gene of *Bifidobacterium breve* 210B. *Appl. Environ. Microbiol.* **76,** 3206-3219 (2010).

Vesth, T., Kagesen, K. & Ussery D. CMG-Biotools, a Free Workbench for Basic Comparative Microbial Genomics. *PLoS ONE* **8**, e60120.

Yi, S.H., Alli, I., Park, K.H. & Lee, B.H. Overexpression and characterization of a novel transgalactosylic and hydrolytic β-galactosidase from a human isolate *Bifidobacterium breve* B24. *New Biotechnol.* **28,** 806-813 (2011).
